# Hypoxic Non-replicating Persistent *Mycobacterium tuberculosis* Develops Thickened Outer Layer That Helps in Restricting Rifampicin Entry

**DOI:** 10.3389/fmicb.2019.02339

**Published:** 2019-10-11

**Authors:** Kishor Jakkala, Parthasarathi Ajitkumar

**Affiliations:** Department of Microbiology and Cell Biology, Indian Institute of Science, Bengaluru, India

**Keywords:** *Mycobacterium tuberculosis*, hypoxia, non-replicating persistence, thick outer layer, polysaccharide, negative charge, rifampicin, permeability

## Abstract

Bacteria undergo adaptive morphological changes to survive under stress conditions. The present work documents the morphological changes in *Mycobacterium tuberculosis* (*Mtb*) cells cultured under hypoxic condition using Wayne’s *in vitro* hypoxia model involving non-replicating persistence stages 1 and 2 (NRP stage 1 and NRP stage 2) and reveals their physiological significance. Transmission electron microscopy of the NRP stage 2 cells showed uneven but thick outer layer (TOL), unlike the evenly thin outer layer of the actively growing mid-log phase (MLP) cells. On the contrary, the saprophytic *Mycobacterium smegmatis* NRP stage 2 cells lacked TOL. Scanning electron microscopy (SEM) and atomic force microscopy (AFM) of the *Mtb* NRP stage 2 cells confirmed the rough uneven surface unlike the smooth surface of the MLP cells. Zeta potential measurements showed high negative charge on the surface of NRP stage 2 cells and polysaccharide specific calcofluor white (CFW) staining of the cells revealed high content of polysaccharide in the TOL. This observation was supported by the real-time PCR data showing high levels of expression of the genes involved in the synthesis of sugars, such as trehalose, mannose and others, which are implicated in polysaccharide synthesis. Experiments to understand the physiological significance of the TOL revealed restricted entry of the biologically low-active 5-carboxyfluorescein-rifampicin (5-FAM-RIF), at concentrations equivalent to microbicidal concentrations of the unconjugated biologically active rifampicin, into the NRP stage 2 cells, unlike in the MLP cells. Further, as expected, mechanical removal of the TOL by mild bead beating or release of the NRP stage 2 cells from hypoxia into normoxia in fresh growth medium also significantly increased 5-FAM-RIF permeability into the NRP stage 2 cells to an extent comparable to that into the MLP cells. Taken together, these observations revealed that *Mtb* cells under hypoxia develop TOL that helps in restricting rifampicin entry, thereby conferring rifampicin tolerance.

## Introduction

*Mycobacterium tuberculosis* (*Mtb*) has emerged as a difficult-to-eradicate pathogen due to its ability to develop drug resistance (Frieden et al., 1993; Morris et al., 1995), to remain dormant in the granuloma in patients and to reactivate itself out of latent infection in immunocompromised and aged individuals (Wells et al., 2007; Kwan and Ernst, 2011). Dormant *Mtb* cells have been found to be recalcitrant to anti-tuberculosis drugs (Iona et al., 2007; Shleeva et al., 2011), making the elimination of the pathogen from granuloma difficult. Many studies have suggested diverse reasons for the recalcitrance of hypoxic *Mtb* cells to antibiotics (Iona et al., 2007; Shleeva et al., 2011), except against metronidazole (Wayne and Sramek, 1994). Many earlier studies have shown diverse types of morphological changes occurring to *Mtb* cells exposed to different types of stress conditions, including hypoxia. Severe cell wall alterations, which increased the proportion of Ziehl-Neelsen (ZN) negative mycobacterial cells, have been found under severe nutritional stress conditions (Nyka, 1974). Cell wall thickening was found in mycobacterial stationary phase induced low oxygen tension cells (Cunningham and Spreadbury, 1998). Altered colony morphology and its correlation with loss of virulence upon continuous subculturing has also been reported (Hunter et al., 2006). Formation of phase-dark ovoid cells (PDOC) with thickened cell walls was observed upon gradual external acidification of the cells (Shleeva et al., 2011).

Cell wall thickening was also observed during the first 3 months of the oxygen reduction in latent mycobacteria (Velayati et al., 2011). In one to 36 months old latent cultures, folding phenomena was observed in 4–10 months of dormancy, spore-like cells by the time of 18 months of dormancy and non-acid-fast cell wall deficient forms in 36 months also been observed (Velayati et al., 2011). Subsequently, the role of wax esters in dormancy (Sirakova et al., 2012), spore like morphotypes in 1 year old broth cultures (Lamont et al., 2012), reduced antibiotic entry in nutrient starved non-replicating condition (Sarathy et al., 2013), more shorter and spherical phenotypes in K^+^ deficient Sauton’s medium (Salina et al., 2014) have also been observed. Apart from this, exopolyphosphatase (ppx2) gene deficient strain of *Mtb* was found to have increased ploy (P) levels, which had a role in increased cell wall thickness and reduced drug permeability (Chuang et al., 2015). Existence of L-form variants, coccoid forms, and granular forms of *Mtb* has been observed under stress conditions (Markova et al., 2012). Mycobacterial cultures were found to have small resting cells under mild nutrient starvation, large resting cells in saline shock starvation (Wu et al., 2016), and cellular swelling upon exposure to sub-inhibitory concentrations of INH (Campanerut-Sá et al., 2016). Recently, deletion of the RD105 region from the clinical strains of mycobacteria led to Rv0071/74 gene fusion with consequential increased cell wall thickening and reduced intracellular antibiotic concentration (Qin et al., 2019).

In the present study, we wanted to document the morphological changes of *Mtb* hypoxic cells and find out whether the morphological changes have any role in the restricted permeability of hypoxic cells to the anti-tuberculosis drug, rifampicin. For this purpose, we used Wayne’s *in vitro* hypoxia model system, with the characteristic non-replicating persistence (NRP) stages 1 and 2 of hypoxic *Mtb* cells (Wayne and Hayes, 1996). Wayne and Hayes postulated that the bacteria recovered from the granulomatous lesions had adopted to oxygen starved environment so that they could not grow on traditional culture methods. To get the bacteria adapt and survive anaerobiosis Wayne has proposed a model where bacteria subjected to self-generated gradual oxygen depletion and enter NRP stage. In this model the morphological changes observed was increased cell length (Wayne and Hayes, 1996). These cells were found to resemble closely the bacilli present in the hypoxic environment encountered by *Mtb* cells in granuloma (Wayne and Hayes, 1996; Aly et al., 2006; Via et al., 2008). In the present study, we examined the morphological changes of hypoxic *Mtb* cells and their contribution to the restricted entry of the anti-tuberculosis drug, rifampicin.

## Materials and Methods

### Bacterial Strains and Growth Conditions

*Mycobacterium tuberculosis* H_37_R_a_ (National JALMA Institute of Leprosy and Other Mycobacterial Diseases, Agra, India; *Mtb*) and *Mycobacterium smegmatis* mc^2^155 (*Msm*) (Snapper et al., 1990) were cultured in Middlebrook 7H9 medium in the presence of 0.2% glycerol and 0.05% Tween 80, with and without ADS (0.5% BSA, 0.75% dextrose and 0.08% NaCl), respectively, at 37°C with shaking at 170 rpm. *M. tuberculosis* H_37_R_a_ cultures were used in biosafety level 2 laboratory. Mid-log phase (MLP) cultures of *Mtb* cells were harvested at OD_600 nm_ ∼0.6.

### Wayne’s *in vitro* Hypoxia Model Setup for *Mtb* and *Msm* Cells

Wayne’s *in vitro* hypoxia model was set up as described (Wayne and Hayes, 1996). In brief, the preparation of the liquid cultures was performed by filling Difco Dubos broth base in 500 ml screw-capped conical flasks up to a level such that, the head space to volume ratio (HSR) was maintained to be 0.5 in the presence 5% glycerol. This was achieved by filling a total of 400 ml of Difco Dubos broth in the screw-capped conical flasks and left a head space of 200 ml above it. ADS was added aseptically to this in a proportion of 10%. ADS addition was performed post-autoclaving and once the media gets cooled down to room temperature. Sterility check was performed by incubating the flasks at 37°C for 24 h with shaking at 170 rpm. One percent of the secondary inoculum from *Mtb* MLP culture was added, and then for proper distribution of the cells, cultures were gently mixed with hand. Subsequently, to obtain a final concentration of 1.5 μg/ml, 1.2 ml of 500 mg/ml methylene blue (Sigma) was added. Flasks were tightly sealed with a white non-serrated precision seal rubber septum (Sigma) and to ensure air tightness flask mouth was tightly wrapped with Parafilm (Bemis NA). Teflon-coated magnetic stirring bars (35 mm) were used with continuous stirring at 130 rpm to prevent settling down of the cells and the flasks were incubated at 37°C. Decolorization of the dye methylene blue was observed as an indicator of oxygen depletion within the cultures as described (Wayne and Hayes, 1996). NRP stage 1 was reached on the day 7 (with mild decolorization of methylene blue) after setting up the hypoxia culture and NRP stage 2 was reached on the day 12 after setting up of the hypoxia culture (with complete decolorization of methylene blue), as described for *Mtb* hypoxia cells (Wayne and Hayes, 1996). A hypoxic system, like that of Wayne’s model for *Mtb* cells, was set up for *Msm* cells also, as described (Dick et al., 1998). The *Msm* cells of NRP stages 1 and 2 were harvested on the day of decolorization of methylene blue, as the indicator, which consistently happened on the days 3 and 10, respectively, after setting up of the hypoxia culture.

### Detection of Dissolved Oxygen in Hypoxia Exposed *Mtb* Cultures Using Oxygen Sensor

A Schott Duran 500 ml volume glass conical flask mouth part was cut and manually it was joined to a 38/40 ground joint socket. A Lutron DO-5509 dissolved oxygen sensor was manually fitted to the male part of the ground joint and the gaps between the sensor and the ground joint was completely sealed with adhesive araldite. This unit was inserted into the ground joint socket of the flask which contained the hypoxia medium and air tightened with vacuum grease. This setup containing the inoculated medium was kept at 37°C with continuous Teflon coated magnetic bar stirring at 130 rpm and monitored the dissolved oxygen at different time points.

### Acid-Fast Staining of *Mtb* Cells

Acid-fast staining of *Mtb* MLP and hypoxic (NRP stages 1 and 2) cells was performed using ZN acid-fast staining kit (HiMedia, India). First a drop of mycobacterial cell suspension was taken from a well shaken culture and spread evenly on a clean glass slide to obtain an even smear. This smear was first heat fixed and then allowed for acid-fast staining as described by the manufacturer of the kit. Few drops of carbol fuchsin were added on to the heat fixed cells and heated over the flame till visual fumes appear. After repeating this step for 3–4 times, heat-fixed slide was washed gently under running tap water. Subsequently, few drops of acid-fast decolorizer were added on the slide and washed immediately under running tap water. Finally, by adding few drops of methylene blue on the decolorized slide, counter staining was performed, and the slide was incubated at room temperature for 30 s. Excess of methylene blue dye was removed by gently washing the slide under running tap water and the slide was air dried. A drop of glycerol was added on the slide and a clean cover slip was mounted carefully without disturbing the cells. The cells were observed under oil immersion and imaged using Carl Zeiss AXIO Imager M1 microscope under 100× objective lens. Axio Vision software (AxioVs40 V 4.8.2.0) was used for image analysis.

### Staining *Mtb* Cells With SYTO-9 and PI

In order to check the viability of the MLP and hypoxic cultures, before they were taken for morphological and ultrastructural analyses, 1 ml of *Mtb* MLP cells and the cells from hypoxia (NRP stages 1 and 2) were pelleted down at room temperature for 10 min at 5000 × *g*. After washing the cell pellet in fresh Middlebrook 7H9 medium, the cell suspension was pelleted down at 5000 × *g* for 10 min at room temperature. The cell pellet was resuspended in 200 μl of fresh Middlebrook 7H9 medium containing 0.2 μl of SYTO-9/PI (propidium iodide) mixture and the supernatant was discarded. Propidium iodide mixture was prepared by mixing 1:1 ratio of SYTO-9/PI of Live/Dead^TM^ *Bac*Light^TM^ Bacterial Viability Kit (Invitrogen) by volume. To prevent direct exposure to light, the Eppendorf tube was covered with aluminum foil and subjected to gentle vortexing for 30 sec. The cell suspension thus obtained was kept for 30 min at 37°C. In parallel, each well in the multi-well glass slide was coated with poly-L-lysine and kept at room temperature for 10 min. Excess poly-L-lysine was removed and ∼4 μl of the SYTO-9/PI incubated cell suspension was added to each well. For proper adhesion of the cells to the poly-L-lysine coated slide, the slide was incubated for 10 min at room temperature in the dark. Subsequently, the slide was gently washed once with fresh Middlebrook 7H9 medium to remove unbound cells. After adding a drop of glycerol on multi-well slide, a clean coverslip was placed, and the cells were observed under oil immersion using Carl Zeiss AXIO Imager M1 microscope under 100× objective lens. Axio Vision software (AxioVs40 V 4.8.2.0) was used for image analysis.

### Scanning Electron Microscopy (SEM)

Sample preparation for SEM was carried out as described (Vijay et al., 2012, 2014a,b), with minor modifications. In brief, 1 ml of *Mtb* MLP cells and cells from hypoxia NRP stages 1 and 2 were harvested by pelleting down at 5000 ×*g* for 10 min at 4°C. Subsequently, the cell pellet was washed once with fresh Middlebrook 7H9 medium and again pelleted down at 5000 ×*g* for 10 min at 4°C. The supernatant was removed, and the cell pellet was resuspended in 500 μl of fresh Middlebrook 7H9 medium. Cell suspension was then fixed with 2% (v/v) glutaraldehyde (Sigma) and incubated in the dark for 20 min at room temperature. Subsequently, the cells were treated with 0.5% osmium tetroxide buffered with 0.15 M sodium cacodylate buffer (Sigma) (pH 7.4) for 2 h at room temperature. Dehydration of the cells was carried out with graded series of ethanol (Merck) (ranging from 20 to 90% with a uniform difference of 20%) and finally washed with 95% ethanol. A drop of the cell suspension was taken on a small piece of silica plate and mounted on an aluminum stub with the help of double-sided carbon tape. These cells were kept overnight in a desiccator to remove excess water content from the sample and then sputter-coated with gold and observed under SIRION scanning electron microscope. Images were captured at 20 kV using TLD detector.

### Transmission Electron Microscopy (TEM)

Sample preparation for transmission electron microscopy was performed as described (Takade et al., 1983; Vijay et al., 2012), with minor modifications. In brief, 1 ml of *Mtb*/*Msm* cells from MLP and the cells from hypoxia (NRP stages 1 and 2) cultures were harvested by pelleting down at 5000 × *g* for 10 min at 4°C. The obtained cell pellet was once again washed with fresh Middlebrook 7H9 medium and the cell suspension was harvested by pelleted down at 4°C for 10 min at 5000 × *g*. The cell pellet was resuspended in 500 μl of fresh Middlebrook 7H9 medium and the supernatant was discarded. 2% (v/v) glutaraldehyde (Sigma) was added and incubated for 20 min at room temperature. Subsequently, the cells were washed with the same buffer and pre-fixed for 1 h at room temperature with 1% (w/v) osmium tetroxide (Sigma) buffered with 0.15 M sodium cacodylate buffer (pH 7.4) (Sigma). The pre-fixed cells were then washed once with the same buffer and re-fixed in 0.15 M sodium cacodylate buffer containing 2% (w/v) tannic acid (Sigma) and 2% (v/v) glutaraldehyde for 2 h at room temperature. Subsequently, the cells were subjected to washing with the same buffer, and then re-fixed in 1% (w/v) osmium tetroxide overnight at 4°C. The cell suspension was dehydrated in a graded series of ethanol solution (Merck) ranging from 20 to 90% with an equal intermittent difference of 20% and finally with 100% ethanol. Cells were infiltrated with 70% LR white resin overnight at 4°C. Subsequently, the cell suspension was subjected to centrifugation at 5000 × *g* for 10 min at 4°C and a part of the cell pellet was added to 100% LR white resin taken separately in a size 1 gelatine capsule (Electron Microscopy Sciences) for embedding. Curing was performed following embedding, at 62°C for 48 h. Subsequently, the gelatine blocks were subjected to trimming using ultramicrotome and 70 nm thickness ultra-thin sections were cut with glass knife. These sections were collected on 150 mesh × 165 μm pitch (Sigma-Aldrich) sized copper grid. The copper grid containing sections were subjected to uranyl acetate staining followed by lead citrate staining as described below.

#### Uranyl Acetate Staining

Uranyl acetate staining was performed by taking a clean Petri plate and a piece of parafilm was attached to the inner side of the plate. In parallel, 1% (w/v) uranyl acetate solution was prepared by dissolving 0.01 g of uranyl acetate powder (Sigma) in 1 ml of 50% methanol (Merck) and a drop of this solution was taken on the parafilm. Subsequently, the grids having ultra-thin sections were kept on the uranyl acetate solution in an inverted manner such that the grids are exposed to uranyl acetate. To prevent direct light exposure, the Petri plate was covered with aluminum foil and incubated at room temperature for 1 h. Grids were then washed with 50% methanol prepared and kept separately in another Petri plate and then subjected to washing with autoclaved de-ionized water. Subsequently, grids were kept at upright position on Whatman No. 1 filter paper overnight after repeating the washing process thrice, following which lead citrate staining was performed.

#### Lead Citrate Staining

Lead citrate (0.4%) was prepared by taking (0.04 g) of lead citrate (Fluka) in 10 ml of boiled water and immediately vortexed vigorously for proper dissolving of lead citrate. Subsequently, for the complete dissolving of the lead citrate, 100 μl of 10 N NaOH (Sigma) solution was added to the 10 ml lead citrate and the mixture was vortexed till it gets dissolved. Few pellets of NaOH was kept in a clean dried Petri dish and was covered with lid for 30 min to block the air contact. Consequently, a drop of lead citrate solution was added on the parafilm and the dish was closed again for 10 min. Subsequently a drop of 0.1% NaOH solution was added in a separate Petri dish and similarly, one more Petri dish was taken with parafilm and added a drop of autoclaved de-ionized water. The copper grid having the ultra-thin sections was kept inverted on the lead citrate solution, which was prepared earlier and immediately it was transferred to the 0.1% NaOH solution for 10 min. Finally, grids were transferred to the fresh water drop which was kept separately in another Petri dish for 10 min. Both the washing steps mentioned above were repeated twice and finally grids were kept overnight on a Whatman No. 1 filter paper in upright position. Grids were observed under transmission electron microscope at 120 kV (BioTwin, FEI).

### Rapid Freeze-Substitution Electron Microscopy of MLP and NRP Stage 2 *Mtb* Cells

Rapid freeze-substitution electron microscopy was performed as mentioned (Yamada et al., 2010). In brief; first, all copper grids were hydrophilized just before the use and 1 μl of highly concentrated MLP (control sample) or NRP stage 2 (experimental sample) bacterial cell pellet was sandwiched between two copper grids on a filter paper. These grids were taken with tweezers and kept in melting propane (Fisher Scientific) for 20 s. Following this, the grids were transferred to liquid nitrogen, detached and immersed immediately in 2% OsO4 (Sigma)/acetone (Merck Millipore) solution and cooled it in freezing ice. These samples were transferred to −85°C freezer for 24 h and gradually brought them to room temperature. These grids were washed 3 times with absolute acetone (Merck Millipore) after discarding OsO4. These grids were treated with a mixture of acetone (Merck Millipore) and epoxy resin (EMS) (1:1) for a day and then transferred to only resin for one more day. Following this, a flat bottom PCR tube was taken and filled with resin and on top of the resin the grids were kept inverted and subjected for polymerization at 70°C for 2 days. The grid was removed, the epoxy bocks were trimmed, and sections were taken at 70 nm thickness using glass knife and stained with uranyl acetate and lead citrate, as described above. These sections were observed under Tecnai Bio-TWIN (at Indian Institute of Science) transmission electron microscope at 120 kV.

### Atomic Force Microscopy (AFM)

Sample preparation for AFM was carried out as described (Bolshakova et al., 2001) with minor modifications. In brief, 1 ml of *Mtb* MLP cells and the equivalent cells from hypoxia (NRP stages 1 and 2) were harvested by pelleting down at 5000 × *g* for 10 min at 4°C. The cell pellet was resuspended in fresh Middlebrook 7H9 medium and was again pelleted down at 4°C for 10 min at 5000 × *g*. The cell pellet was resuspended in 200 μl of fresh Middlebrook 7H9 medium. In parallel, a drop of this bacterial cell suspension was placed on a clean glass coverslip and air-dried for 10 min at room temperature. Subsequently, the coverslip was washed gently twice with de-ionized water following which the cells were once again subjected to air drying at room temperature for 10 min. The coverslip was then placed on an aluminum stub with the help of double-sided carbon tape as an adhesive. Images were captured by keeping the microscopic cantilever in non-contact mode.

### Zeta Potential – Cell Surface Charge Measurements

Sample preparation for zeta potential was carried out as described (Wilson et al., 2001; Ayala-Torres et al., 2014), with minor modifications. Initially, 1 ml of the *Mtb* MLP cells and cells from hypoxia (NRP stages 1 and 2) cultures were pelleted down at room temperature for 10 min at 5000 × *g* and the cell pellets were washed once with fresh Middlebrook 7H9 medium. The cell suspension was again subjected to centrifugation at 5000 × *g* for 10 min at room temperature and the cell pellet was resuspended in 1 ml of 1× PBS. Subsequently, 1 ml of the cell suspension was taken into a disposable polystyrene low volume cuvette and cell surface charge detection was carried out using Zeta sizer nano series (Nano-ZS90; Malvern Instruments).

### Zetasizer – Cell Size Measurements

Sample preparation for Zetasizer cell size measurements were carried out as described (Wilson et al., 2001; Ayala-Torres et al., 2014), with minor modifications. Initially, 1 ml of the *Mtb* MLP cells and equivalent cells from hypoxia (NRP stages 1 and 2) cultures were harvested by pelleting down the cells at 5000 × *g* at 4°C for 10 min. The cell pellet was washed once with fresh Middlebrook 7H9 medium and the supernatant was discarded. Subsequently the cell suspension was again subjected to centrifugation at 5000 × *g* for 10 min at 4°C. The cell pellet was resuspended in 1 ml of fresh Middlebrook 7H9 medium and was subjected to cell size measurements using Zeta sizer nano series (Nano-ZS90, Malvern Instruments).

### 5-FAM-Rifampicin Permeability Assay

The conjugation of 5-carboxy fluorescein (5-FAM) to rifampicin to get 5-FAM-rifampicin (5-FAM-RIF) was custom made, as described (Sebastian, 2016). The 5-FAM-RIF molecule has maximum excitation at 488 nm and maximum emission at 519 nm. The obtained 5-FAM-RIF fluorescence property was used to estimate the antibiotic entry into MLP as well as into NRP (NRP stages 1 and 2) cells before and after bead beating. 5-FAM-RIF entry experiment was performed as described below. Four ml of the *Mtb* MLP culture and cells from hypoxia (NRP II) were harvested by pelleting down at room temperature for 10 min at 5000 × *g*. The cell pellet was processed in two different ways, one with washing and the other without washing. In the case of protocol involving washing, the cell pellet washed once with fresh Middlebrook 7H9 medium and then it was subjected again to centrifugation at room temperature for 10 min at 5000 × *g* following which the supernatant was discarded. The cell pellet, without washing or after washing, was resuspended in 20 ml fresh Middlebrook 7H9 medium. Ten microliter of 3 mg/ml 5-FAM-RIF conjugate was added to the cell suspension to obtain a final concentration of 1.5 μg/ml (which is equivalent to 1 μg/ml rifampicin in terms of biocidal activity). The resultant cell suspension was incubated at 37°C with 170 rpm agitation. Five hundred microliter aliquots of 5-FAM-RIF exposed culture was taken at 0, 40, and 120 min, and processed in two different ways, one with a quick wash with 500 μl of fresh ice-cold Middlebrook 7H9 broth once and another without washing. The flow cytometry analysis was performed using BD FACS Canto system (for the unwashed sample) or BD FACS Verse (for the washed sample) with a 488 nm solid state laser and a 527/32 nm emission filter (GFP) at low or medium flow rate. 208 (FSC), 333 (SSC) were the photomultiplier tube (PMT) voltage settings. For instrument calibration, FACSuite cytometry set up and tracking (CS&T, Becton Dickinson) beads were used. FACSuite software was used for flow cytometry data analysis. At every time point, 50,000 cells were gated. As the control, the auto-fluorescence values of the samples before 5-FAM-RIF addition (0 min) were used and the median of the auto fluorescence for GFP filter was kept at 2-log_10_ fluorescence units for *Mtb* MLP cells and NRP stage 2 cells individually.

### Calcofluor White (CFW) Staining of Mycobacterial Cells for Microscopy

Calcofluor white (CFW) staining of *Mtb* cells for microscopy was carried out as described (Maeda and Ishida, 1967; Wood, 1980). One ml of the mycobacterial culture from MLP and NRP stage II cells were pelleted down at 4°C for 10 min at 5000 × *g* and the cell pellet was washed once with fresh Middlebrook 7H9 medium. Subsequently, the cell suspension was pelleted down at 4°C for 10 min at 5000 × *g*. Cell pellet was resuspended in 500 μl of fresh Middlebrook 7H9 medium. 0.1% solution of CFW (Fluka) at a dilution of 1:100, was added to the cell suspension and incubated at 37°C for 1 h. In parallel, a clean multi-well slide was coated with 4 μl of poly-L-lysine for 10 min and the excess poly-L-lysine was discarded. For proper adherence of the cells, the cell suspension was incubated at room temperature for 10 min on the poly-L-lysine coated slide, after which the excess of poly-L-lysine was discarded. Subsequently, the wells were washed thrice with 1× PBS gently using pipetman and air-dried for 10 min. With a drop of glycerol, a clean coverslip was mounted on the multi-well slide and observed under oil immersion using Carl Zeiss Axio Imager M1 microscope at 100× objective lens using DAPI fluorescence. The Axio Vision software was used for image analysis and editing.

### Release of NRP Stage 2 Cells From Hypoxia Into Normoxia

Hypoxia-exposed *Mtb* (NRP stages 1 and 2) cultures were released from hypoxia and harvested by pelleting down at 5000 × *g* for 10 min at room temperature. The cell pellet was resuspended in fresh 1 ml Middlebrook 7H9 medium. The resuspension was inoculated into 100 ml of fresh Middlebrook 7H9 medium and incubated at 37°C with shaking at 170 rpm.

### Fourier Transform Infrared Spectroscopy (FTIR)

*Mtb* cells from both MLP (from 4 × 100 ml culture) and NRP stage 2 (4 × 400 ml culture) were harvested and the outer capsular layer was isolated to detect their compositional differences using Fourier Transform Infrared spectroscopic analysis. The capsular polysaccharide isolation from hypoxia (NRP stage 2) as well as from MLP was performed as described (Ortalo-Magne et al., 1995; Daffe and Laneelle, 2001). In brief, 400 ml (4 × 100 ml) of the MLP and 1600 ml (4 × 400 ml) of hypoxia (NRP stage 2) cultures were harvested by centrifuging them at 5000 × *g* for 10 min at room temperature. To the cell pellet, acid-washed, pre-sterilized, 4 mm (diameter) solid soda lime glass beads (Sigma) were added at 1:5 proportion (wet cell weight: glass beads). The cell pellet-glass bead mixture was subjected to 30 sec to 1 min gentle shaking and to this two ml of the fresh Middlebrook 7H9 medium was added and again subjected for gentle shaking. After bead beating, the cell suspension was separated from the glass beads using pipetman and the beads were washed twice with 2 ml fresh Middlebrook 7H9 medium. The supernatant from each wash was collected and was filtered through Whatman No. 1 filter paper, and the filtrate was concentrated to 1/10th of its original volume using a rotatory evaporator (Thermo savant) by keeping the drying rate at medium level. Six volumes of 95% ethanol (Merck) was added to this concentrated solution, mixed gently with pipetman and the suspension was incubated overnight at 4°C. After overnight incubation, the suspension was centrifuged at 4°C for 1 h at 14,000 × *g*, and the pellet was dissolved in 1/10th of the original volume with autoclaved double-distilled water. Six volumes of the ice-cold ethanol were added to the cell suspension and again subjected to centrifugation process as mentioned earlier. Above mentioned process was repeated one more time and finally, the pellet was dissolved in 1/10th of the original volume with autoclaved double-distilled water and dialyzed using 3 kDa snake skin pleated dialysis tubing (Pierce) against autoclaved double-distilled water at 4°C for 3 days to remove the excess glycerol content. Rotatory evaporator was used to concentrate the dialyzate by keeping the drying rate of the instrument at medium level. To the concentrated dialyzate, chloroform and methanol were added in such a way that the homogeneous phase has a proportion of chloroform/methanol/water in the ratio of 1:2:0.8 (v/v/v). The obtained suspension was mixed and allowed to stand for 1 h at room temperature for partitioning of the solvents. Finally, one volume of chloroform and one volume of water were added to this mixture, mixed well and waited till the phase separation occurred. Subsequently, the aqueous, organic and the interphases were recovered individually. By keeping the drying rate of the instrument at medium level the aqueous upper phase was concentrated using rotatory evaporator. To obtain glucan-rich solution, interphase was extracted thrice with autoclaved double-distilled water, and all the three extracts were later pooled. To the concentrate of aqueous phase and to the pooled extract of the interphase, six volumes of ice-cold ethanol was added. The suspensions were kept at 4°C overnight, following which they were subjected to centrifugation at 14,000 × *g*, at 4°C for 1 h. The pellet was resuspended in 1/10^th^ of the original volume in autoclaved double-distilled water and the supernatant was discarded. The steps from the addition of six volumes of ethanol for dialysis against autoclaved double distilled water at 4°C for 1–3 days were repeated. Using a rotatory evaporator, by keeping the drying rate of the instrument at medium level, the resultant dialyzate was concentrated. The concentrated dialyzate was resuspended in HPLC grade methanol and, using rotatory evaporator and by keeping the drying rate at medium level, the methanol suspension was evaporated. Finally, FTIR analysis was performed for the polysaccharide powder thus obtained. Prior to sample analysis using FTIR, a control was run without the addition of any polysaccharide material and the peak so obtained was used for normalization.

### Preparation of Cells for Total RNA Isolation

To understand the transcriptome profile differences between *Mtb* MLP and NRP stage 2, cells from both the samples were harvested. Firstly, *Mtb* cultures (4 × 400 ml) exposed to hypoxia were removed from the hypoxia chamber and kept on ice with minimal disturbance for 30 min to arrest the cells at their actual metabolic state. In parallel, GSA centrifuge cups were also kept under ice, and the centrifuge (Beckman Coulter Avanti) with rotor (AG-2506) was kept for pre-cooling at 4°C for 30 min. After 30 min, the culture from the ice-chilled flasks was released from hypoxia by aseptically removing the rubber septa, and immediately transferred to pre-cooled GSA cups. This cell suspension was centrifuged in pre-cooled rotor at 10,000 × *g* for 10 min at 4°C. After centrifugation, the cell pellet was resuspended in 1 ml ice cooled fresh Middlebrook 7H9 medium and the supernatant was discarded. The cell suspension was pelleted again in pre-chilled rotor at 10,000 × *g* for 10 min at 4°C. Finally, the supernatant was removed, and the pellet was snap-frozen in liquid nitrogen and stored at −70°C. In the case of MLP cells, 100 ml of MLP culture was taken and the remaining procedure for cell culture harvesting was followed as in the case of hypoxia cultures.

### Total RNA Preparation From *M. tuberculosis* Cells

Total RNA was isolated using hot phenol method (Wecker, 1959; Ausubel and Kingston, 1987), with slight modifications. *M. tuberculosis* cell pellets from MLP and NRP stage 2 were taken from −70°C and lysed using micro pestles for ∼30 min with intermittent snap-freezing and thawing. After cell lysis, 1 ml of the lysis buffer (3 M sodium acetate 40 μl, 0.5 M EDTA 24 μl, 20 mM VRC 30 μl, 1% SDS 120 μl, DEPC-treated water 986 μl) was added to the lysed cells. The tubes were mixed gently by inversion immediately after the addition of the lysis buffer. Subsequently, two fresh Eppendorf tubes were taken and 500 μl each of the cell suspension was distributed into both the tubes. Following this, 500 μl of hot phenol, which was already kept at 60°C heating block, were added to both the tubes. After phenol addition, the tubes were kept at 60°C in heating block for 10 min. To ensure complete homogenous mixing, at every 2 min interval the suspension was mixed by inverting the tubes for at least 5 times. After 10 min incubation at 60°C, the tubes were kept on ice for 5 min. Subsequently, the lysate was subjected to centrifugation at 4°C for 10 min at 8000 × *g*. The aqueous phase was collected into a fresh tube after centrifugation, and it was subjected again for hot phenol extraction twice more. To the final aqueous phase, equal volume of ice-cold phenol:chloroform mixture (1:1, v/v) was added, and the contents were mixed 3–4 times gently by inverting the tubes. The homogenous mixture thus obtained was subjected to centrifugation at 4°C for 10 min at 8000 × *g*. The aqueous phase was collected and again equal volume of ice-cold phenol:chloroform mixture (1:1) was added and the contents were mixed 3–4 times gently by inversion of the tube and subjected to centrifugation at 4°C for 10 min at 8000 × *g*. Into a fresh tube, the aqueous phase was collected and to this, 1/10th volume of 0.3 M sodium acetate (pH-5.2) was added and mixed using pipetman till homogenous mixture obtained. Following this, 2.5 volume of 95% ice-cold ethanol (Merck) was added and mixed gently by inverting the tubes and then kept for RNA precipitation at −70°C, overnight. After thawing the solution, the RNA was pelleted down by centrifugation at 4°C for 20 min at 8000 × *g*. The supernatant was removed, and the pellet was resuspended in 800 μl of 80% ethanol (Merck) and subjected to centrifugation at 4°C for 10 min at 8000 × *g*. The obtained supernatant was discarded and the 80% ethanol washing process was repeated once more. The pellet was air-dried for 15 min at room temperature and dissolved in 40 μl of DEPC-treated water. The integrity of the total RNA preparation was verified using formaldehyde-agarose gel electrophoresis, as described (Ausubel and Kingston, 1987). The presence of undegraded (without streak) 23S, 16S, and 4S bands of RNA was confirmed. Some contamination from genomic DNA could be observed at times. The RNA was quantitated using NanoDrop^TM^ 1000 Spectrophotometer (Thermo Fisher Scientific) and stored at −70°C.

### DNase I Treatment of Total RNA

In order to remove DNA contamination, if any, the RNA samples were treated with 10 units of DNase I (Thermo Fisher Scientific) per 10 μg of total RNA at 37°C for 40 min. Subsequently, after transferring tubes to ice, 200 μl DEPC water was added and subsequently equal volumes of phenol (Tris-HCl saturated phenol, pH 7.4): chloroform mixture (1:1) was added. The tubes were mixed well by inversion to ensure that complete homogeneous mixture has formed. The mixture was centrifuged at 4°C for 10 min at 8000 × *g*. The aqueous phase was collected into fresh tube and 1/10th volume of 0.3 M sodium acetate (pH 5.2) was added and mixed well, following which 2.5 volumes of ice-cold ethanol (Merck) was added and mixed well by inverting the tube. The RNA was set for precipitation at −70°C overnight. After overnight incubation at −70°C, tubes were thawed and the RNA was pelleted at 4°C for 20 min at 8000 × *g*, following which the supernatant was discarded. The RNA pellet was suspended in 400 μl of 80% ethanol and again subjected to centrifugation at 4°C for 10 min at 8000 × *g*. Post-centrifugation, the supernatant was discarded and the washing step with 80% ethanol was repeated. Finally, the RNA pellet was air dried at room temperature for 15 min and dissolved in 20 μl of DEPC water and quantitated using NanoDrop^TM^ 1000 Spectrophotometer (Thermo Fisher Scientific) and stored at −70°C. The integrity of the total RNA preparation was verified using formaldehyde-agarose gel electrophoresis (Ausubel and Kingston, 1987). The presence of undegraded (without streak) 23S, 16S, and 4S bands of the total RNA sample was confirmed using formaldehyde agarose gel electrophoresis.

### cDNA Preparation

Initially 100 ng of DNase I treated RNA was taken for each gene. To this RNA, 10 pico moles of the gene specific reverse primers (Sigma), 50 μM dNTP mix (Thermo Fisher Scientific), 20 units of RiboLock RNase inhibitor (Thermo Fisher Scientific), 200 units of RevertAid-Premium Reverse Transcriptase (RNaseH minus, thermostable), 5× RT buffer were added and the final volume was made up to 20 μl by adding filter-sterilized double-distilled water. Initial denaturation was performed at 65°C for 5 min, annealing at 56°C for 30 min and the final enzyme inactivation at 95°C for 10 min. The cDNA was stored at −20°C and used for real time PCR analysis. cDNA of *sigA* from the same sample was used as normalization control.

### Real Time PCR

Real Time PCR was performed according to the guidelines and protocols provided along with the Real Time PCR EvaGreen Mastermix (GBiosciences). All the experiments were performed according to comparative ΔΔCt method (Wang et al., 2006). Ten microliter of EvaGreen qPCR Mastermix-ROX (G Biosciences), 10 pmoles of gene specific forward and reverse primers each (Sigma) and 2 μl of cDNA were added to make up to 20 μl of reaction volume with double-distilled water. For all the selected genes, experiment was performed in biological duplicate and technical triplicates were made for each biological set. With each real time experiment, *sigA* was used as the normalization control in each plate. Reactions were performed by keeping the parameters as follows: initial denaturation at 95°C for 5 min, amplification with 40 cycles of three stage amplification, 95°C for 10 s, annealing for 20 s at 56°C and amplification at 72°C for 20 sec. To check the specificity of the primers and respective formation of specific product, melt curve was also performed for all the genes. CFX96 Real Time PCR system from Bio-Rad was used for the Real Time experiment. Comparative Ct (ΔΔCt) method was used for the calculation of fold change in the expression levels of mRNA. cDNAs obtained from mRNAs of *Mtb* MLP and NRP stage II cells were used for Real Time PCR experiment where the expression levels of MLP were used as the control samples.

## Results

### Experimental Rationale and Strategy

The studies were performed on *Mtb* cells in the non-replicating persistent stages 1 and 2 (NRP stage 1 and NRP stage 2), in comparison to MLP cells, as described (Wayne and Hayes, 1996). The morphology characteristics of *Mtb* NRP stage 1 and NRP stage 2 cells were compared with those of the actively growing cells from MLP cultures of *Mtb*. The *Mtb* cells reached NRP stages 1 and 2 on the days 7 and 12, which was indicated by the decolorization of methylene blue due to oxygen depletion in the hypoxia culture (Wayne and Hayes, 1996). The morphological changes were documented, and the physiological advantage conferred by the specific changes for the survival of *Mtb* cells against the anti-tuberculosis antibiotic, rifampicin, was determined. *M. smegmatis* cells at NRP stages 1 and 2, as described by Dick et al. (1998), and *Mtb* MLP cultures (OD_600 nm_ ∼ 0.6) were used as the control samples.

### Dissolved Oxygen Levels in *Mtb* Hypoxic Cultures

**FIGURE 1:**
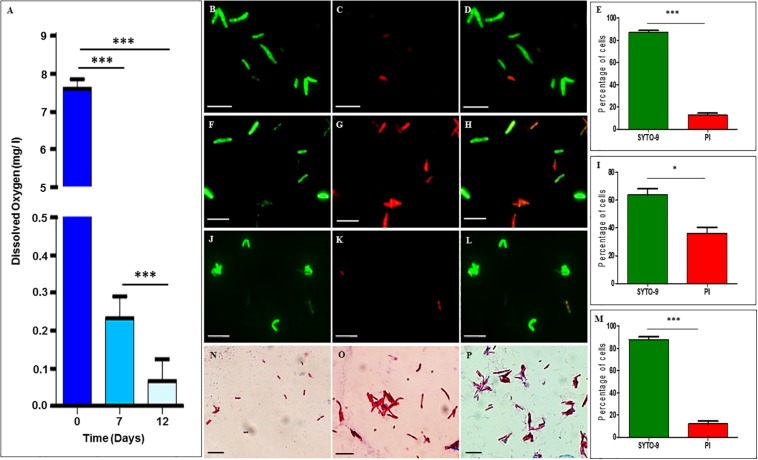
Dissolved oxygen levels in and SYTO-9/PI staining of Mtb cells from MLP, and NRP stages 1 and 2. **(A)** Levels of dissolved oxygen in the hypoxic cultures at different days. **(B–E)** NRP stage 1 cells stained with: **(B)** SYTO-9, **(C)** PI, **(D)** merged image of SYTO9-positive and SYTO-9/PI-positive cells, and **(E)** percentage of SYTO-9-positive and PI-positive cells (*n* = 3055). **(F–I)** NRP stage 2 cells stained with: **(F)** SYTO-9, **(G)** PI, **(H)** merged image of SYTO9-positive and SYTO-9/PI-positive cells, and **(I)** percentage of SYTO-9-positive and PI-positive cells (*n* = 3188). **(J–M)** MLP cells stained with: **(J)** SYTO-9, **(K)** PI, **(L)** merged image of SYTO9-positive and SYTO-9/PI positive cells, and **(M)** percentage of SYTO-9-positive and PI-positive cells (*n* = 1845). **(N–P)** Ziehl-Neelsen staining of *Mtb* cells: **(N)** MLP, **(O)** NRP stage 1, and **(P)** NRP stage 2 cells. Scale bar for all the images is 5 μm. Statistical significance was calculated using Student’s *t*-test. ^∗^*p* ≤ 0.05; ^∗∗∗^*p* ≤ 0.0005.

The dissolved oxygen levels in the cultures of *Mtb* NRP stages 1 and 2 cells, as measured using the oxygen sensor, Lutron DO-5509 dissolved oxygen meter, showed dissolved oxygen of 7.6 ± 0.23 mg/L on day 0, which reduced to 0.23 ± 0.05 mg/L on day 7 (NRP stage 1) and to 0.067 ± 0.05 mg/L on day 12 (NRP stage 2) (Figure 1A). The partial decolorization of methylene blue was observed on day 7 and complete decolorization on day 12, as reported (Wayne and Hayes, 1996). This indicated that the decolorization of methylene blue, which is an indication of hypoxia setting in Wayne and Hayes (1996), was indeed due to reduction in the dissolved oxygen levels in the medium. After confirming that hypoxic atmosphere has set in in the cultures, we examined the extent of viability of the cells of NRP stages 1 and 2, in comparison to that of the MLP cells, using SYTO9/PI staining.

### Viability and Purity of the *Mtb* Cells in Hypoxic Cultures

SYTO-9/propidium iodide staining showed that ∼87.18 ± 1.52% and ∼63.80 ± 4.38% of *Mtb* cells from NRP 1 and 2 stages, respectively, were found to be SYTO9-positive and hence viable (Figures 1B–I, respectively). The remaining 12.81 ± 1.52% and ∼36.19 ± 4.38% of the cells of NRP stage 1 and NRP stage 2, respectively, were either propidium iodide-positive and/or SYTO9 and PI-positive. As expected, ∼87.97 ± 2.31% of the MLP *Mtb* cells were SYTO-9 positive while the remaining were either PI-positive and/or SYTO9 & PI-positive (Figures 1J–M). Compared to the cells of NRP stage 1, even though the percentage of the SYTO-9 positive cells in the NRP stage 2 were about 20% less, majority of the cells in both these stages were SYTO-9 positive and hence taken to be viable. All the experiments using NRP stage 2 cells, were cultured in hypoxia flasks where the head space ratios (HSR) were strictly maintained at 0.5 by keeping the surface to volume ratio 0.08 cm^2^/ml, as instructed (Wayne and Hayes, 1996). We ensured the integrity and purity of the *Mtb* NRP stage 1 and NRP stage 2 cells by ZN staining as well, which showed that the majority of the cells of the NRP stage 1 and NRP stage 2 were ZN-positive, like in the case of the MLP cells (Figures 1N–P).

### Cell-Length Heterogeneity of *Mtb* NRP Stage 1 and Stage 2 Cells

**FIGURE 2:**
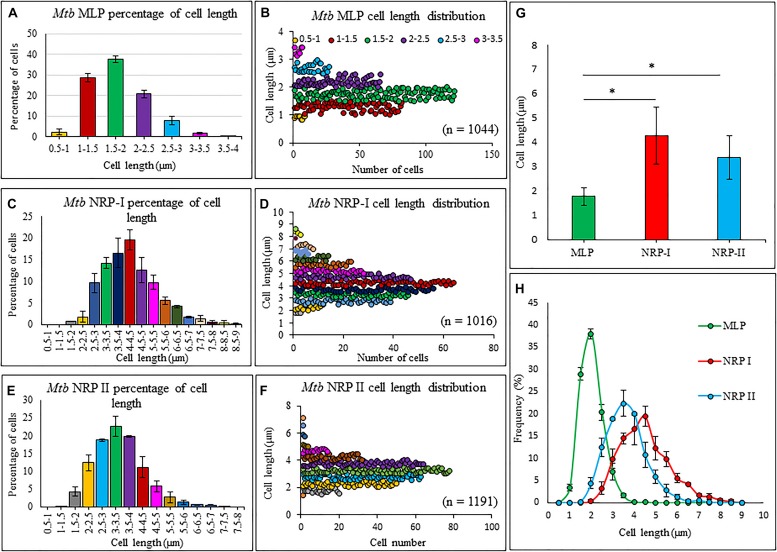
Length measurements and the distribution of *Mtb* MLP, NRP stages1 and 2 cells. Proportion of cells of specific lengths of: **(A)** *Mtb*, **(C)** *Mtb* NRP stage 1 cells, and **(E)** *Mtb* NRP stage 2 cells. Length distribution of: **(B)** MLP, **(D)** NRP 1, and **(F)** NRP 2 cells. **(G)** Comparison of cell-lengths. **(H)** Comparison of cell-length distribution. Statistical significance was calculated using Student’s *t*-test. ^∗^*p* ≤ 0.05.

The average length of the *Mtb* NRP stage 1 and stage 2 cells, found by length measurements of the DIC images using microscopy and zetasizer based on dynamic light scattering, were significantly higher than that of MLP cells (Figure 2 and Supplementary Figure S1, respectively). The increase in length might have been due to the possibility of cell elongation during nucleoid replication and segregation, prior to cell division. This possibility is strengthened by the report that *Mtb* NRP stage 2 cells were found to have completed DNA replication (prior to which cell elongates) but not cell division (Wayne and Hayes, 1996). The cell-length heterogeneity of *Mtb* NRP stage 2 cells was not as prominent as in the case of actively growing MLP population of the cells, reported by us earlier (Vijay et al., 2017).

### *Mtb* NRP Stage 1 and Stage 2 Cells Possess Rough Unevenly Wrinkled Surface

**FIGURE 3:**
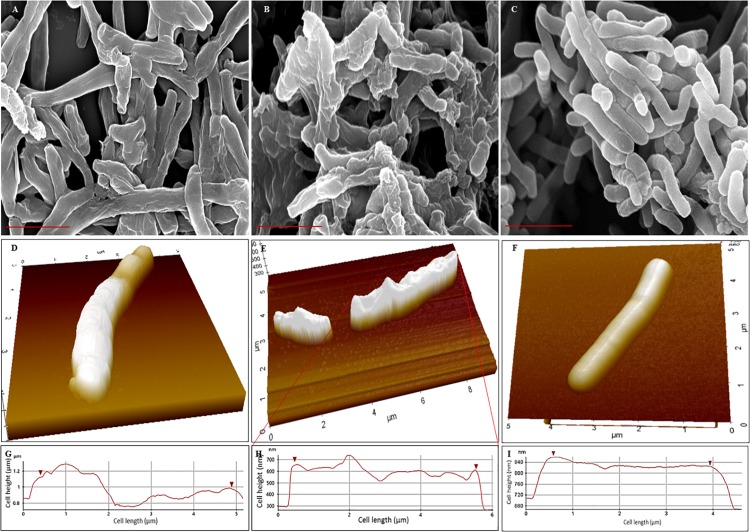
Scanning electron micrographs and Atomic force micrographs of *Mtb* NRP stage 1, stage 2, and MLP cells. **(A–C)** Scanning electron micrographs of: **(A)** NRP stage 1 cells with wrinkled roughness on their outer surface; **(B)** NRP stage 2 cells with increased wrinkled roughness on outer surface; **(C)** *Mtb* MLP cells with smooth outer surface. Scale bar is 2 μm. **(D–F)** Atomic force micrographs of: **(D)** NRP stage 1 cell with wrinkled rough surface; **(E)** NRP stage 2 cell with highly wrinkled rough surface; the image shows a normal-sized and a short-sized cell; **(F)** *Mtb* MLP cell with smooth surface. **(G–I)** The scan of the surface of NRP stage 1 cell (*n* = 74), NRP stage 2 cell (*n* = 111), and MLP cell (*n* = 49), respectively, during AFM analysis showing the differences in the wrinkledness of the surface of the respective cell.

Scanning electron microscopy (SEM) revealed that the surface of NRP stage 1 cells was unevenly wrinkled and rough which considerably increased in the NRP stage 2 cells (Figures 3A,B). The MLP cells possessed clear smooth surface (Figure 3C). It was likely that the rough and unevenly wrinkled surface of the NRP stages 1 and 2 cells might have been a consequence of the serial dehydration process with methanol performed during sample preparation for SEM. In order to rule out this possibility, the surface of the cells from the MLP, NRP stages 1 and 2 were examined using AFM in the contact independent mode of scanning.

AFM analyses of the cells showed that NRP stage 1 cells (*n* = 74) and NRP stage 2 cells (*n* = 111) do possess unevenly wrinkled rough surface, with the stage 2 cells having increased roughness and wrinkles (Figures 3D,E). On the contrary, the surface of MLP cells (*n* = 49) were even and smooth (Figure 3F). The line profiles of the surface of the cells of NRP stages 1 and 2 and of MLP correlated well with the morphological features of wrinkles and roughness of the former and the smooth surface of the latter, respectively (Figures 3G–I). The observations from AFM supported the results from SEM, confirming that the NRP stage 1 and NRP stage 2 cells have developed an altered morphology in response to hypoxic stress, unlike the MLP cells growing under normoxic condition.

### Cells of NRP Stages 1 and 2 Possess Thick Outer Layer

**FIGURE 4:**
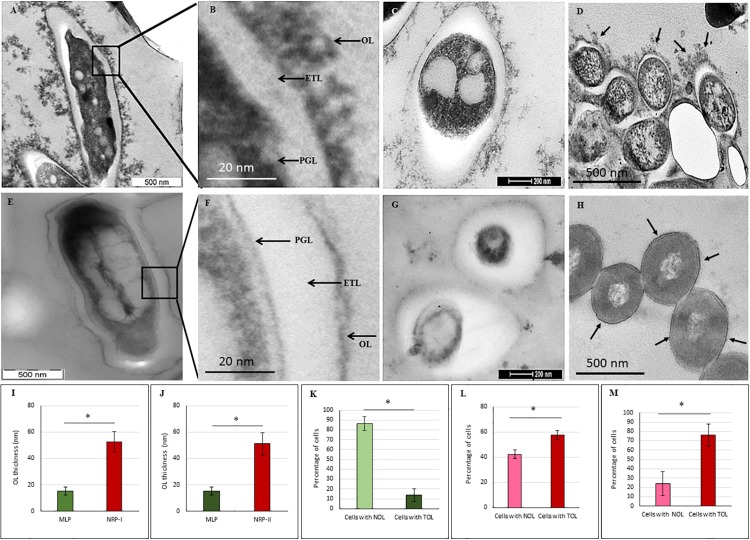
Transmission electron micrographs of *Mtb* NRP stage 2 and MLP cells. **(A,B)** Images of the longitudinal section of NRP stage 2 cell and its higher magnification showing a clear peptidoglycan layer (PGL), electron transparent layer (ETL), and a thick outer layer (TOL). **(C)** Transverse sections of the NRP stage 2 cell (*n* = 138). **(D)** Transverse section of the NRP stage 2 cell obtained using freeze substitution method. **(E**,**F)** Image of the longitudinal section of MLP cell and its higher magnification showing a clear peptidoglycan layer (PGL), electron transparent layer (ETL), and a thin normal outer layer (NOL). **(G,H)** Images of the transverse sections of the MLP cells (*n* = 58) using: **(G)** conventional TEM and **(H)** freeze-substitution methods. **(I,J)** Outer layer thickness of NRP stage 1, NRP stage 2 and MLP cells. Outer layer thickness of: **(I)** MLP and NRP stage 1 cells (*n* = 97); **(J)** MLP and NRP stage 2 cells. **(K–M)** Percentage of cells with NOL and TOL. Proportion of NOL and TOL containing cells in: **(K)** MLP, **(L)** NRP stage 1, and **(M)** NRP stage 2. Arrowhead indicates the outer layer of the cells. Statistical significance was calculated using Student’s *t*-test. ^∗^*p* ≤ 0.05.

Transmission electron micrographs (TEM) of NRP stage 2 cells (*n* = 138) in the longitudinal and transverse sections revealed strikingly uneven, rough and loosely bound thick outer layer (TOL), unlike that of the MLP cells (*n* = 58) (Figures 4A–C,E–G, respectively). However, the morphologies of peptidoglycan layer (PGL) and electron transparent layer (ETL) of NRP stage 2 cells were like those of MLP cells (Figures 4A–C,E–G, respectively), which were consistent with the profiles reported earlier (Rastogi et al., 1986; Takade et al., 2003; Vijay et al., 2012). Thus, the NRP stage 2 cells have an altered surface under hypoxic stress, unlike the MLP cells under normoxia. The morphology of the NRP stage 1 cells (*n* = 97) seemed to be an intermediary stage in the alteration of morphological features during the transition of growth conditions from normoxia to hypoxia.

The average thickness of the normal outer layer (NOL) of the MLP cells, measured at multiple locations, was found to be 15.17 ± 3.07 nm (Figures 4E,I,J). Therefore, the cells with OL thickness ≤15.17 ± 3.07 nm were considered to have NOL and the cells with OL thickness >15.17 ± 3.07 nm were taken to possess TOL. According to this cut-off, the thickness of TOL of NRP stage 2 cells was 51.14 ± 8.45 nm and that of NRP stage 1 cells was 52.59 ± 7.73 nm (Figures 4I,J). While 86.3 ± 7.02% of the MLP cells showed NOL, 13.66 ± 6.28% of the MLP cells possessed TOL (Figure 4K). On the contrary, 57.65 ± 3.51% of the NRP stage 1 cells showed TOL, the remaining 42.34 ± 3.51% cells possessed NOL (Figure 4L). The proportion of the cells with TOL increased to 76 ± 11.62% in the NRP stage 2 population, while the remaining 24 ± 13% of the cells possessed NOL (Figure 4M). Thus, the NRP stage 1 cells, which are at a stage of transition from the MLP to the NRP stage 2 (Aly et al., 2006), reflected the transition in the development of TOL also, from the MLP cells to the NRP stage 2 cells (Figure 4L). To avoid the possibility as to whether the reagent used to fix the cells (i.e., OsO4) at 4°C (see transmission electron microscopy section under Materials and Methods) has affected the cell morphology, the presence of loose TOL was further confirmed using rapid-freeze substitution electron microscopy of NRP stage 2 and MLP cells (Figures 4D,H, respectively). Further, the loose TOL of NRP stage 2 cells observed in TEM correlated well with the rough unevenly wrinkled surface of the cells observed under SEM and AFM.

**FIGURE 5:**
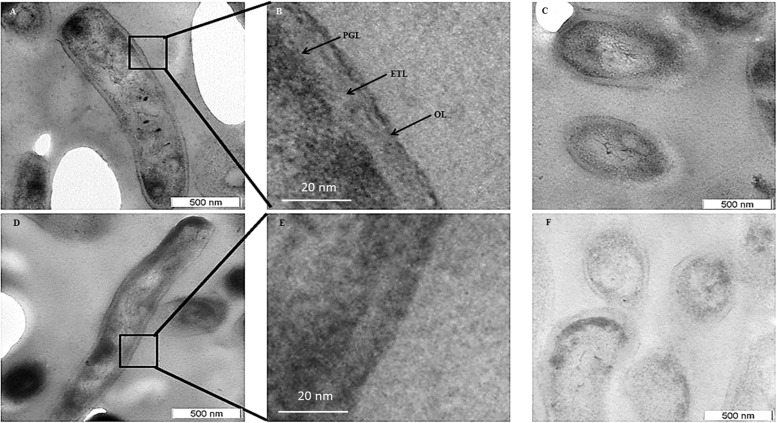
Transmission electron micrographs of *M. smegmatis* MLP and NRP stage 2 cells. **(A,B)** Images of the longitudinal section of NRP stage 2 cell and its higher magnification showing a clear peptidoglycan layer (PGL), electron transparent layer (ETL), and a thin outer layer (OL). **(C)** Images of the transverse section of the NRP stage 2 cells. **(D,E)** Images of the longitudinal section of MLP cell and its respective higher magnification showing a clear peptidoglycan layer (PGL), electron transparent layer (ETL), and a thin outer layer (OL). **(F)** Images of the transverse sections of MLP cells.

The cells of the saprophytic species, *Mycobacterium smegmatis* (*Msm*), also go through NRP stages 1 and 2 (on the days 5 and 8, respectively, post-decolorization of methylene blue) in response to hypoxia, as described (Dick et al., 1998). However, in comparison, the *Msm* NRP stage 2 cells, which were also processed for TEM in a manner identical to that used for *Mtb* NRP stage 2 cells, did not possess TOL (Figures 5A–C). The MLP *Msm* cells (control sample) possessed the classical PGL, electron transparent layer (ETL), and a smooth and thin outer layer (OL), as reported earlier by others and us (Daffe et al., 1989; Vijay et al., 2012; Figures 5D–F). This indicated that the response of the *Msm* to hypoxia does not involve outer layer thickening, although several other features of NRP stage 2 cells of *Msm* and *Mtb* cells were reported to be comparable (Dick et al., 1998).

### TOL Contains Negatively Charged Polysaccharides

**FIGURE 6:**
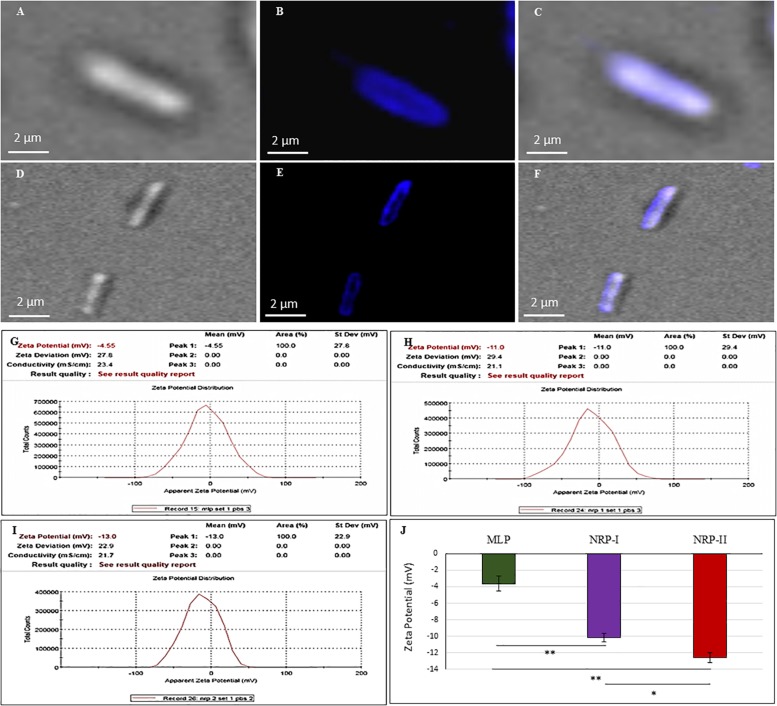
Calcofluor White (CFW; polysaccharide specific) staining of *Mtb* NRP stage 2 and MLP cells. **(A–C)** Images of NRP stage 2 cell under: **(A)** DIC; **(B)** CFW fluorescence; **(C)** merged images. **(D–F)** Images of MLP cell under: **(D)** DIC; **(E)** CFW fluorescence; **(F)** merged images. **(G–J)** Cell surface charge analysis of *Mtb* MLP, NRP stage 1, and stage 2 cells by Zeta potential analyzer. Zeta potential value of: **(G)** MLP cells; **(H)** NRP stage 1 cells; **(I)** NRP stage 2 cells. **(J)** Comparative zeta potential values of the MLP, NRP stage 1, and stage 2 cells. Statistical significance was calculated using Student’s *t*-test. ^∗^*p* ≤ 0.05; ^∗∗^*p* ≤ 0.005.

Staining of NRP stage 2 *Mtb* cells with CFW, which specifically binds β (1→4) glucopyranose in polysaccharides (Maeda and Ishida, 1967; Wood, 1980), showed the presence of β (1→4) glucopyranose-rich polysaccharides in TOL (Figures 6A–C). The MLP cells also showed the presence of β (1→4) glucopyranose-rich polysaccharides (Figures 6D–F) indicating that the OL in MLP cells is also composed of such polysaccharides, as shown by other groups earlier in mycobacteria (Rastogi et al., 1986; Ortalo-Magne et al., 1995). Therefore, it was likely that NRP stage 2 cells might possess higher levels of such polysaccharides in TOL, as compared to their normal levels in the NOL in the actively growing MLP cells.

Zeta potential is the electrical potential of the interfacial region between the aqueous environment and the bacterial surface (Wilson et al., 2001; Ayala-Torres et al., 2014). This can be estimated by measuring the cellular electrophoretic mobility in an electric field (Wilson et al., 2001; Ayala-Torres et al., 2014). While the MLP cells had a zeta potential value of ∼ (−) 3.625 ± 0.9 mV, the NRP stages 1 and 2 cells showed ∼ (−) 10.17 ± 0.52 mV and ∼ (−) 12.57 ± 0.58 mV, respectively (Figures 6G–J). Thus, as the cells proceeded from MLP to NRP stages 1 and 2, the net negative charge on the cell surface increased, indicating increased content of negatively charged polysaccharides on the cell surface as part of the TOL.

### Chemical Nature of the Functional Groups in the TOL Polysaccharides

**FIGURE 7:**
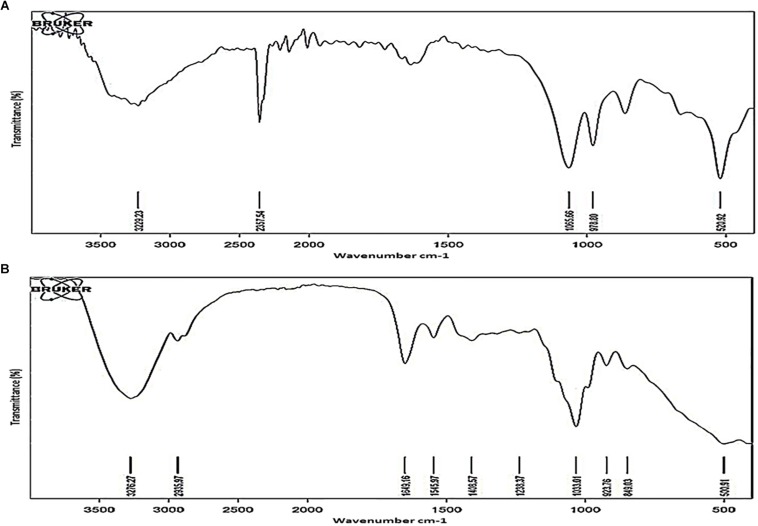
FTIR spectra of outer layer polysaccharides purified from *Mtb* MLP and NRP stage 2 cells. FTIR spectra of the outer layer polysaccharides purified from: **(A)** MLP cells and **(B)** NRP stage 2 cells.

Polysaccharides were purified from TOL and NOL of the NRP stage 2 cells and the MLP cells, as reported (Ortalo-Magne et al., 1995; Daffe and Laneelle, 2001), and the chemical groups present in them were analyzed using Fourier Transform Infrared (FTIR) spectroscopy, as described (Pavia et al., 2001). The major functional group differences in the polysaccharides of TOL and NOL were distinct (Figure 7). Specific transmittance value of 1649 cm^–1^ showed the presence of carbonyl group (C = O) and the specific transmittance value of 1545 cm^–1^ showed its bonding with amino group (Supplementary Figure S2A), and not with carboxylic, aldehyde, or ketone groups (Supplementary Figure S2B). These results indicated that TOL contains molecules involved in the interaction between carbonyl group (C = O) containing molecules (probably sugars) with the amide group containing molecules (probably proteins). Since the carbonyl and amine groups contain electronegative atoms (Supplementary Figure S2C), the presence of compounds with CO-NH linkage in the TOL can confer negative charge to the cells containing TOL.

**FIGURE 8:**
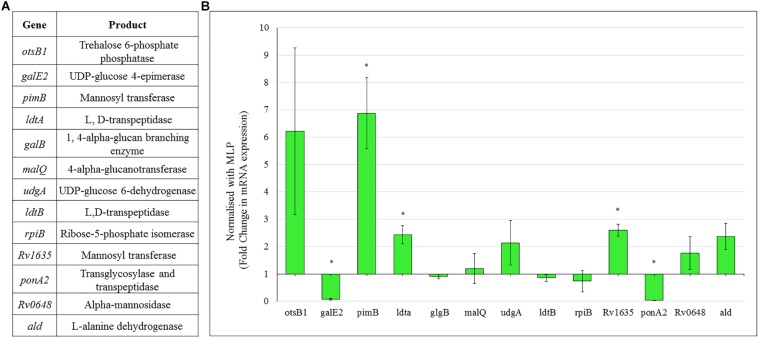
Real time PCR data for the genes involved in polysaccharide metabolism in *Mtb* NRP stage 2 cells. **(A)** Genes and their products for whose expression was analyzed using qPCR. **(B)** Expression profile of the genes involved in trehalose and outer capsular polysaccharide biosynthesis. Quantitative RT-PCR was performed using the primers listed in Supplementary Table S1.

Consistent with the high levels of polysaccharides in the TOL, the expression of genes coding for the enzymes involved in the metabolism of sugars, such as trehalose and mannose, were found to be high in the *Mtb* NRP stage 2 cells (Figure 8). Consistent with the possibility that the levels of trehalose might be high in TOL, qPCR analysis showed ∼6-fold higher levels of expression of trehalose-6-phosphate phosphatase (otsB1), a key biosynthetic enzyme that converts trehalose-6-phosphate to free trehalose in mycobacteria (De Smet et al., 2000).

### Physiological Significance of TOL in the NRP Stage 2 Cells

**FIGURE 9:**
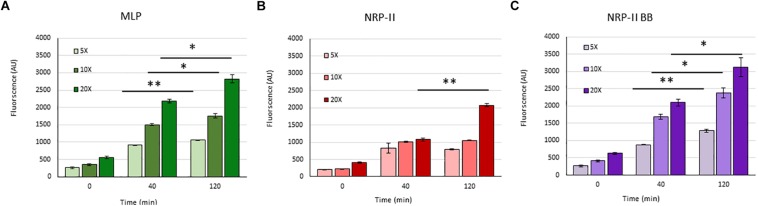
Quantitation of the flow cytometry profile of the entry of 5×, 10×, and 20× MBC rifampicin-equivalent of 5-FAM-RIF into MLP and NRP stage 2 cells at different time points. Arbitrary values of 5-FAM fluorescence at 0, 40, and 120 min in: **(A)** MLP cells, **(B)** NRP stage 2 cells, and **(C)** after bead beating of the NRP stage 2 cells. Statistical significance was calculated using Student’s *t*-test. ^∗^*p* ≤ 0.05; ^∗∗^*p* ≤ 0.005.

**FIGURE 10:**
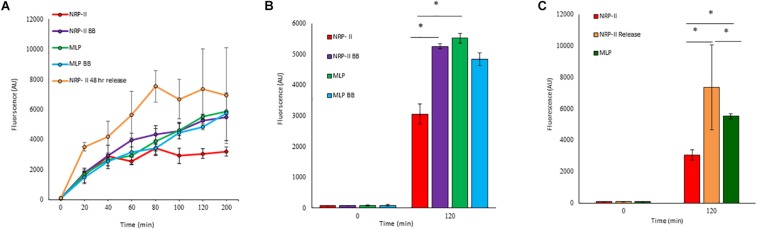
Quantitation of flow cytometric analyses of the permeability of 10× MBC rifampicin-equivalent of 5-FAM-RIF into the washed *Mtb* MLP and NRP stage 2 cells before and after bead beating, and after release from hypoxia into normoxia. **(A)** Time course of the increase of 5-FAM-RIF fluorescence in the MLP and NRP stage 2 cells, before and after bead beating and from 48 h after release from hypoxia into normoxia. **(B)** Quantitation of the extent of fluorescence in the MLP and NRP stage 2 cells, before and after bead beating. **(C)** Levels of 5-FAM-RIF fluorescence in the MLP, NRP stage 2 hypoxia cells and in the NRP stage 2 cells at 0 and 2 h after 48 h post-release from hypoxia into normoxia, upon incubation with 5-FAM-RIF for 0 min (at 48 h, immediately upon release from hypoxia into normoxia) and 120 min (at 120 min after 48 h post-release from hypoxia into normoxia). Statistical significance was calculated using Student’s *t*-test. ^∗^*p* ≤ 0.05.

Presuming that the TOL might have a role in restricting permeability, we studied the extent of entry of 5-carboxy-fluorescein-rifampicin (5-carboxy-fluorescein conjugated to rifampicin, 5-FAM-RIF) into NRP stage 2 cells, in comparison with that into MLP cells (Supplementary Figure S3). 5-FAM-RIF, of quantity equivalent to 10× MBC rifampicin, showed only ∼2.5% bioactivity and hence did not affect cell viability during the antibiotic entry assay (Sebastian, 2016). We did not use radioactively labeled rifampicin as the lethality would have restricted the permeability analysis of the rifampicin-exposed cells. Unwashed MLP and NRP stage 2 cells were exposed to different concentrations (equivalent of 5×, 10×, and 20× MBC rifampicin) of 5-FAM-RIF. Monitoring the fluorescence at 0 min, 40 min and at 120 min, during the exposure, showed increased 5-FAM fluorescence in both the MLP and NRP stage 2 cells over time (Figures 9A,B and Supplementary Figures S4A,B). Significant increase in the fluorescence of 5-FAM-RIF, which was comparable to that of MLP cells, was observed when TOL in the unwashed NRP stage 2 cells was removed by gentle bead beating, as described (Ortalo-Magne et al., 1995; Figure 9C and Supplementary Figure S4C). Experiments using briefly washed NRP stage 2 cells (see section Materials and Methods under 5-FAM-rifampicin permeability assay) also showed similar profile when monitored over 200 min (Figure 10A). The fluorescence in the MLP cells continued to increase steadily but not in the NRP stage 2 cells (Figure 10A). The 5-FAM-RIF fluorescence at initial time points in the NRP stage 2 cells might have been due to 24% of the cells having NOL (see Figure 4M). The comparable profile of 5-FAM-RIF fluorescence in the unwashed and briefly washed NRP stage 2 cells showed that the brief washing did not affect the TOL and hence the permeability to 5-FAM-RIF. The enhanced fluorescence of 5-FAM-RIF in the bead beaten NRP stage 2 cells indicated that the removal of TOL might have facilitated increased permeability of 5-FAM-RIF into the NRP stage 2 cells (Figures 10A,B). The increase in the extent of 5-FAM fluorescence was also verified at 48 h after the release of the NRP stage 2 cells from hypoxia into normoxia to achieve natural removal of the TOL. This was achieved by monitoring the NRP stage 2 cells, at 48 h post-release into normoxia, by growing in fresh Middlebrook 7H9 medium under shaking at 170 rpm, followed by incubation with 5-FAM-RIF for 0 min and 120 min. Substantially increased fluorescence of 5-FAM-RIF was found in the cells after bead beating and at 120 min post-release (Figures 10A,C and Supplementary Figures S5B–D). It was significantly higher than that in the NRP stage 2 cells, but comparable to the fluorescence in the MLP cells (Figures 10A,C and Supplementary Figures S5A,E). This observation indicated that that the NRP stage 2 cells, upon release into normoxia, might have lost TOL and gained the normal thickness for the outer layer (NOL). This decrease in the thickness of OL from TOL to NOL might have enhanced the permeability of 5-FAM-RIF. All these observations confirmed that the TOL in the hypoxic *Mtb* NRP stage 2 cells significantly hindered 5-FAM-RIF entry indicating that the development of TOL in the NRP stage 2 cells has a physiological role in restricting rifampicin entry.

## Discussion

### The Presence of Thick Outer Layer in the *Mtb* NRP Stage 2 Cells Established Using Different Methods of Sample Preparation

Our study reveals that thickening of outer layer, with the accumulation of high levels of negatively charged molecules, confers the ability on *Mtb* cells to restrict the entry of rifampicin. Scanning, transmission and AFM confirmed highly altered cell surface and thickened outer layer of *Mtb* NRP stage 2 cells. The proportion of ZN negative mycobacterial cells have been found to increase due to mycobacterial cell wall alterations under severe nutritional stress conditions (Nyka, 1974). Further, it was also shown that ZN-negative specimens represent *Mtb* cells in dormant state (Seiler et al., 2003). However, unlike in these studies, where the cells had been starved for a long duration, the ZN-positivity of the NRP stage 2 cells showed that they have not been under nutritionally starved condition. The ability of NRP stage 2 cells to initiate cell division, upon release into normoxia (Wayne and Hayes, 1996), might be due to this possibility. Even though the uneven nature of TOL could be argued to be due to preparation of the transmission electron microscopy samples, the confirmation of rough uneven surface by AFM gives credibility to the observations for the following reasons. AFM has the following advantages: (i). live, unprocessed, unfixed cells can be analyzed at a resolution of fraction of a nanometer; (ii) gives three-dimensional topography of cells at higher resolutions. Above all, the presence of the loosely bound TOL in the NRP stage 2 cells was confirmed using rapid freeze-substitution method, a very different method for the preparation of the samples.

For the determination of the ultrastructure of TOL of NRP stage 2 *Mtb* cells, we essentially used two methods, one involving the conventional method of preparation of the samples (Takade et al., 1983) and the other using the rapid freeze-substitution method (Yamada et al., 2010), which differs from the conventional method of in the preparation of the samples. Both these methods have been successfully used to study the cell envelope ultrastructure of mycobacteria by many groups. Although these two methods of preparation of the samples have allowed similar overall conclusions to be drawn in all the studies, they revealed specific and conspicuous differences between the ultrastructure of mycobacterial cell envelope due to the inherent differences in the procedures between the two methods to which the fluid-like properties of the lipid-rich cell envelope have not been equally amenable for analysis. Therefore, we first used the well-established conventional method of sample preparation (Takade et al., 1983), which had been successfully used to study the cell wall outer layer thickening in the stationary phase mycobacterial cells (Cunningham and Spreadbury, 1998), to distinguish between resistant and non-resistant strains (Velayati et al., 2009), and cultures of 150 days from the cultures at the onset of stationary phase (Shleeva et al., 2011), and to characterize exopolyphosphatase deficient *Mtb* strains (Chuang et al., 2015). This conventional method of the preparation of samples for TEM gave clear conspicuous images of the OL, PGL, ETL and cell membrane in our earlier studies on the ultrastructure of cell wall layers of *M. tuberculosis* and *M. smegmatis* (Vijay et al., 2012). In the present study also, this conventional method of preparation of the samples showed clear images of the loosely bound TOL. Nevertheless, to ensure that the TOL was not decreased due to any reason during the conventional method of sample preparation and thereby to further confirm the results obtained using the conventional method of preparation, we used rapid freeze-substitution method, as described (Hobot et al., 1985; Hunter and Beveridge, 2005; Yamada et al., 2010; McDonald and Webb, 2011), which also gave identical results thereby the conclusions derived from the conventional method remained unchanged.

In fact, the observations we made earlier using the conventional method of preparation of the samples for TEM revealed clear single layer of the OL (Vijay et al., 2012). However, instead of a single-layered OL, a bi-layered architecture was revealed by cryo-electron tomography (CET) (Hoffmann et al., 2008) and cryo-electron microscopy of vitreous sections (CEMOVIS) (Zuber et al., 2008). CET or CEMOVIS studies have not contradicted or disproved the observations made using the traditional method for the preparation of samples for TEM. However, these methods gave a detailed architecture of only the OL, as a bi-layered membrane consisting of lipids, but not of the architecture of the inner layers, PGL and ETL. It was mentioned that the layers in between the plasma membrane (PM) and the outer membrane (OM), designated as L1 and L2, by CET could not be assigned to any envelope layer by structural appearance alone (Hoffmann et al., 2008). They proposed that these layers might be related to the peptidoglycan and arabinogalactan-mycolate network and that their visualization by CET was difficult. However, the topology of PGL and ETL, which were revealed by the conventional preparation of samples, could not be obtained clearly using CET or CEMOVIS. Thus, as seen by the conventional method, the study accepted general organization of mycobacterial cell envelope that includes bi-layered PM, PGL, arabinogalactan-mycolate ETL, and OM.

A study using CEMOVIS commented that the absence of ETL in their preparations might probably be due to the presence of lipids, which could not be detected by the CEMOVIS method (Zuber et al., 2008). Although another explanation given was that its presence in the conventional TEM might be due to an artifact of the method, it has been shown that ETZ disappears when mycolic acid synthesis is impaired either by mutation in *M. smegmatis* (Wang et al., 2000) or by treatment with INH in *Mycobacterium avium* (Mdluli et al., 1998), thereby confirming that ETL/ETZ is not an artifact arising from the conventional TEM preparations. Further, they suggested that staining with OsO_4_ led to the loss of the bilayer nature of OM (Zuber et al., 2008). This could be the reason that the OM was found as a single layer, and not as a bilayer in the conventional method of sample preparation, where OsO_4_ is used for membrane staining, like in our present study. Using CEMOVIS of *M. smegmatis tmptB* mutant, which cannot synthesize glycopeptidolipids, and *Corynebacterium glutamicum* mutant, which cannot synthesize mycolic acid, the same group has shown that a part of the OM is made up of mycolic acid in *C. glutamicum* and glycopeptidolipids in *M. smegmatis* (Zuber et al., 2008). It was of interest to note that using conventional method of preparation of *M. smegmatis* mc^2^155 and *M. smegmatis* TM99 mutant, which is impaired in the synthesis of glycopeptidolipids, Etienne et al. (2002) have shown that a part of the OL is composed of glycopeptidolipids. Thus, both conventional method and CEMOVIS analysis led to the same conclusions, with respect to the glycopeptidolipids forming a part of the OM. Thus, the architecture of mycobacterial cell wall revealed by the conventional TEM and CET and CEMOVIS gave comparable results, but showed differences in the details probably due to the differential amenability of the fluid-like property of the lipid-rich cell envelope to the differences in the procedures for the sample preparations in the conventional method as compared to those in CET and CEMOVIS. In a similar manner, in the present study, the analysis of TOL using conventional method of preparation of the sample and rapid freeze-substitution method gave identical conclusions, with both the images showing thickened outer layer in the NRP stage 2 *Mtb* cells but not in the MLP cells.

### Heterogeneity in the Extent of Morphological Alterations in *Mtb* NRP Stage 2 Cells

The length distribution of NRP stages 1 and 2 cells showed heterogeneity, like in the case of MLP cells. However, the average length of the NRP stages 1 and 2 cells were found to be significantly higher than that of MLP cells (see Figures 2G,H). The increase in the average lengths of NRP stages 1 and 2 cells was consistent with the fact that the NRP stages 1 and 2 cells were at a stage after nucleoid replication and segregation, necessitating doubling of cell length, but prior to division (Wayne and Hayes, 1996). Further, the proportion of 57.65 ± 3.51% of the NRP stage 1 cells possessing TOL increased to 76 ± 11.62% in the NRP stage 2 population, with the remaining 24 ± 13% of the cells possessed NOL (see Figures 4L,M). Thus, all the cells in the NRP stage 2 did not possess TOL. A possibility for the lack of TOL in all the cells of NRP stage 2 could be due to heterogeneity in the metabolic status of the source culture (MLP), a hallmark of mycobacterial populations (Vijay et al., 2017; reviewed in Dhar et al., 2016). Due to the heterogeneity in the metabolic status, all the cells in the MLP might not have responded in the same way to the hypoxic stress. Consistency in the proportions of cells with TOL and NOL in the NRP stage 2 cells in biological triplicates ruled out the possibility of the loosely bound TOL getting erratically detached from the cells during sample preparation for TEM. The presence of TOL in a significantly and consistently high proportion of NRP stage 2 cells rules out any artifact in the preparation of samples for TEM. Nevertheless, the interesting observation is the consistent presence of TOL in 13.66 ± 6.28% of the MLP cells (see Figure 4K). This raises the question as to what is the role of TOL in a small proportion of the actively growing MLP cells? It will be a matter of concern in tuberculosis treatment if such subpopulations exist in the patients. It is possible that the existence of such subpopulations in tuberculosis patients could be one of the many reasons for the requirement for the prolonged treatment of patients with microbicidal concentrations of drugs.

### The Probable Role of TOL Components in the Rifampicin Entry Restriction

Since both the MLP cells and the NRP stage 2 cells showed the presence of β (1→4) glucopyranose-rich polysaccharides, it was likely that the polysaccharide content of NRP stage 2 cells in the TOL might be much higher than the levels in the NOL of the MLP cells. This possibility was supported by the observation that as the cells proceeded from MLP to NRP stages 1 and 2, the net negative charge on the cell surface increased, indicating increased content of negatively charged polysaccharides on the cell surface as part of the TOL. Another possibility was that more diverse types of negatively charged polysaccharides would have accumulated in the TOL. Polyhydroxyl groups in trehalose have been found to form hydrogen bonds between carbohydrate and protein (Barton, 2005). Trehalose is also known to protect bacterial cells against desiccation under severe stress conditions, including hypoxia (Chen and Haddad, 2004; reviewed in Arguelles, 2000; Elbein et al., 2003). Reduced cellular levels of trehalose dimycolates (TDMs) have been found to increase sensitivity to multiple antibiotics widely used for antibacterial chemotherapy (Nguyen et al., 2005). Further, the higher negative charge, and thereby the hydrophilicity conferred by the negative charge, of TOL components of the NRP stage 2 cells could be interpreted to be enabling the cells to restrict entry of relatively non-polar molecule, rifampicin. However, an integrated study using Wayne’s *in vitro* hypoxia model at pH 5.8 showed that this need not be the case (Piccaro et al., 2015). They have demonstrated that the drugs of non-polar nature, namely rifampicin, rifapentine, bedaquiline, clofazimine, and nitrazoxamide could effectively reduce cfu of hypoxic cells by ≥ 2-log_10_. However, contrary to the expectation, the drugs of hydrophilic nature, namely metronidazole, moxifloxacin, pyrazinamide, ethambutol, isoniazid, and meropenem could not appreciably reduce the cfu of hypoxic cells (Piccaro et al., 2015). Therefore, the polar, hydrophilic nature of the TOL need not be a factor that might facilitate restriction in the entry of the non-polar (lipophilic) rifampicin. These observations suggest that multiple factors such as the increased physical thickness and negative charge may be contributing to the restricted entry of rifampicin into *Mtb* NRP stage 2 cells with TOL.

### Would TOL Restrict the Permeability of Other Antibiotics Also?

We confined our experiments using rifampicin only and not using other antibiotics as it was not possible to use radioactively labeled antibiotics since they would still be bacteriocidal in action, which would have hampered permeability assays due to cell killing. Further, fluorophore conjugated antibiotics was not a possibility for other antibiotics, unlike we got for rifampicin. Nevertheless, it is quite likely that the observed thickened capsular outer layer may not be antibiotic specific. Rather, it most probably would show a similar reduction in the entry for most of the lipophilic antibiotic molecules for the following reasons. The envelope layer present even in the MLP mycobacterial cells acts as a permeability barrier to rifampicin, which was observed in 1970’s (Hui et al., 1977). Later, Brennan and Nikaido reported that because the peptidoglycan sacculus is covered by arabinogalactan layer and since these layers are hydrophilic layers, they may prevent the entry of hydrophobic molecules (Brennan and Nikaido, 1995). Liu et al. suggested that these two layers, which are covalently linked to outer mycolic acid layer, forms waxy non-fluidic barrier restricting the entry of both hydrophobic and hydrophilic molecules (Liu et al., 1995). Subsequently, it was also shown by the same group that mutants defective in mycolate biosynthesis showed increased uptake and sensitivity to chloramphenicol, erythromycin, rifampicin and novobiocin (Liu and Nikaido, 1999). Transposon mutagenesis studies with the genes involved in mycolic acid biosynthesis also showed increased penetration and sensitivity to ciprofloxacin, pyrazinamide, isoniazid and rifampicin (Gao et al., 2003; Singh et al., 2003, 2005). Apart from these, it was shown that the permeability of the polysaccharide layer to lipophilic probes is about 2 orders of magnitude lesser than that shown by the conventional phospholipid bilayer (Plesiat and Nikaido, 1992). Altered LPS chains with O chains showing reduced antibiotic uptake has also been observed (Robbins et al., 2001). In view of all these observations, it is quite likely that the observed thickened capsular outer layer may not be a strategy shown by *Mtb* cells against rifampicin only.

### Relevance of the Study of TOL in *Mtb* H_37_R_a_ Cells

We used the avirulent *Mtb* H_37_R_a_, which has dysregulated *dosS/dosR* regulon expression as well mutated transcriptional regulator PhoP, as the experimental system instead of the virulent *Mtb* H_37_R_v_. PhoP is a regulator required for the biosynthesis of cell envelope lipids and many adaptation pathways, including *dosS/dosR* regulon in *Mtb* (Pérez et al., 2001; Chesne-Seck et al., 2008; Frigui et al., 2008; Gonzalo-Asensio et al., 2008; Lee et al., 2008; Ryndak et al., 2008). Hence it could be argued that the dysregulated *dosS/dosR* regulon expression and the non-functional PhoP in *Mtb* H_37_R_a_ may affect hypoxic responses. However, recent studies reported that the ultrastructure of the multidrug (MDR) and extensively drug resistant (XDR) mycobacteria, despite presumably having functional PhoP and intact *dosS/dosR* regulon expression also showed a marked difference in the thickness of the cell wall compared to the susceptible mycobacterial isolates (Velayati et al., 2009). In a subsequent study of prolonged hypoxia-exposed (for 18 months) H_37_R_v_ also revealed an increase in the cell wall thickness (Velayati et al., 2011). The identical response of *Mtb* H_37_R_v_ and *Mtb* H_37_R_a_ in developing TOL under hypoxic condition show that *dosS/dosR* and PhoP regulons may not have a major role in the development of TOL. Another possibility is that alternate pathways may exist even if PhoP is non-functional. Apart from this, our molecular level data also showed an increased expression of the genes involved in polysaccharide biosynthesis, compared to the unexposed MLP cells, which seemed to have majorly contributed to the development of outer layer thickness in NRP stage 2 cells. The observed molecular level changes in our study are also correlating with the earlier observed gene expression level studies of *M. tuberculosis* H_37_R_v_ under Wayne’s *in vitro* hypoxia model (Voskuil et al., 2004; Tudo et al., 2010). Further, despite having PhoP regulon and *dosS/dosR* gene systems, *Msm* cells do not develop thickened OL under hypoxia. Further, it has been shown that the hypoxic response of *Msm* is comparable to that of *Mtb* H_37_R_v_ (Dick et al., 1998), despite which *Mtb* alone develops TOL but not *Msm*. These observations justify the use of *Mtb* H_37_R_a_ as an experimental system for the study of hypoxia response of *Mtb*.

### Outer Layer Thickening in Other Mycobacterial Species Under Hypoxia

*Mtb* and other mycobacterial cells have been found to undergo morphological changes involving cell wall thickening when exposed to different types of stress conditions, including hypoxia induced under a variety of culture conditions other than Wayne’s model. Six-months old stationary phase cultures of *Mtb* H_37_R_v_ and *Mycobacterium bovis* BCG (*Mbo* BCG), suffering from hypoxia, have been found to develop TOL of ∼16–21 nm thickness, with the *Mtb* cells showing higher thickness (Cunningham and Spreadbury, 1998). Even 1-month old anaerobic *Mtb* cultures, suffering from hypoxia due to growth under mineral oil also showed thickened outer layer. The thickness of the OL of these *Mtb* and *Mbo* BCG hypoxic cells was about 2.5-fold lesser than the thickness of the OL of *Mtb* cells grown by us using Wayne’s model. Thus, the responses of *Mtb* and *Mbo* BCG cells to hypoxia have similarities (Lim et al., 1999). On the contrary, the *Msm* cells cultured under anaerobic condition even for 35 days did not show TOL but showed lysis (Cunningham and Spreadbury, 1998). We have also found that the NRP stages of *Msm* cells do not develop TOL. However, we found that *Msm* NRP stage 2 cells (day 8) were intact. Thus, the response of *Msm* species to hypoxia is different from the response of *Mtb* and *Mbo* BCG cells to hypoxia. However, several other features of NRP stages of *Msm* cells, such as synchronous division upon release into normoxia, replication initiation after first cell division, and resistivity to ofloxacin, were reported to be similar to those shown by *Mtb* (Dick et al., 1998). Interestingly, hypoxic cultures of *Mycobacterium aurum* (*Mau*) were viable without decrease in cfu upto 16 days, subsequent to which there was drastic decline in cfu (Sood et al., 2016). This indicated that the response of *Mau* species to hypoxia seems to be different from that of *Mtb* and *Mbo* BCG, but probably similar to that of *Msm*, which also showed lysis in 35 days (Cunningham and Spreadbury, 1998).

### Cell Wall Thickening in Mycobacteria Under Other Stress Conditions

Cell wall thickening can be induced by several other stress conditions as well. Gradual external acidification of *Mtb* cells showed the formation of PDOC with thickened cell wall within 150 days after the onset of stationary phase (Shleeva et al., 2011). *Mtb* H_37_R_v_ cells, which were kept stirred at low rpm in sealed tubes for prolonged periods showed different types of morphological changes (Velayati et al., 2011). While development of folds was observed in 4–10 months of culture, spore-like cells were found by 18 months, and loss of acid-fastness and cell wall deficiency were observed in 36 months (Velayati et al., 2011). Spore-like morphotypes were found in 1-year old broth cultures of *Mycobacterium avium* subsp. paratuberculosis (Lamont et al., 2012). Besides these observations, it was reported that deficiency in exopolyphosphatase (ppx2) gene led to accumulation of polyphosphate [poly (P)], which in turn caused increase in cell wall thickness thereby reducing drug permeability (Chuang et al., 2015).

### Broader Role for Negatively Charged Polysaccharide Outer Layer in Bacteria

Many bacterial systems, with mycobacteria being no exception, develop polyanionic polysaccharide outer layer called capsules for antiphagocytosis, as bacterial decoy for antimicrobial peptides, for pathogenesis, colonization of respiratory tract, cellular invasion and so on Daffe and Etienne (1999), Stokes et al. (2004), Llobet et al. (2008). The present study shows that the pathogenic species of mycobacteria, but not saprophytic mycobacteria, also use similar strategy to develop negatively charged TOL in response to hypoxia that helps in restricting rifampicin entry. Thus, the development of thickened outer layer is used by *Mtb* cells as an yet another strategy to protect themselves from antibiotics, and thereby continue to survive in the presence of antibiotics, during hypoxic stress. The TOL might be able to prevent entry of other solutes also and might give a desiccative effect to the cells, especially due to the presence of trehalose (Elbein et al., 2003; Chen and Haddad, 2004). Once the cells come out of hypoxia into normoxia, the TOL gets removed and normal morphology is attained.

## Data Availability Statement

The raw data supporting the conclusions of this manuscript will be made available by the authors, without undue reservation, to any qualified researcher.

## Author Contributions

PA and KJ designed the experiments, analyzed the data, and wrote and read the manuscript. KJ performed the experiments. PA contributed reagents, materials, and analysis tools.

## Conflict of Interest

The authors declare that the research was conducted in the absence of any commercial or financial relationships that could be construed as a potential conflict of interest.
