# Supplementary material for: Hypoxic Non-replicating Persistent Mycobacterium tuberculosis Develops Thickened Outer Layer That Helps in Restricting Rifampicin Entry
Source: Front Microbiol. 2019 Oct 11;10:2339. doi: 10.3389/fmicb.2019.02339 (PMC6797554; doi:10.3389/fmicb.2019.02339)
Supplement: Supplementary file 1 [file Data_Sheet_1.PDF]

Supplementary Material

Hypoxic Nonreplicating Persistent *Mycobacterium tuberculosis* Develops Thickened Outer Layer that helps in Restricting Rifampicin Entry

Kishor Jakkala, Parthasarathi Ajitkumar*

Department of Microbiology and Cell Biology, Indian Institute of Science, Bangalore, Karnataka, India.

*Corresponding author. Parthasarathi Ajitkumar, Tel: 91-80-2293-2344; E-mail: ajitkpartha@gmail.com

This document contains:

- Supplementary Figures (S1 – S5)
- Supplementary Table S1

A

NRP-II	
Cell Size (µm)	Percentage of cells (%)
1.9	0.6
2.3	3.4
2.6	8.9
3.09	14.8
3.5	18.6
4.1	19.6
4.8	18.1
5.5	12.2
6.4	3.8

B

MLP	
Cell Size (µm)	Percentage of cells (%)
1.9	12.5
2.3	27.2
2.6	31.1
3.09	21.7
3.5	7.5

C

D

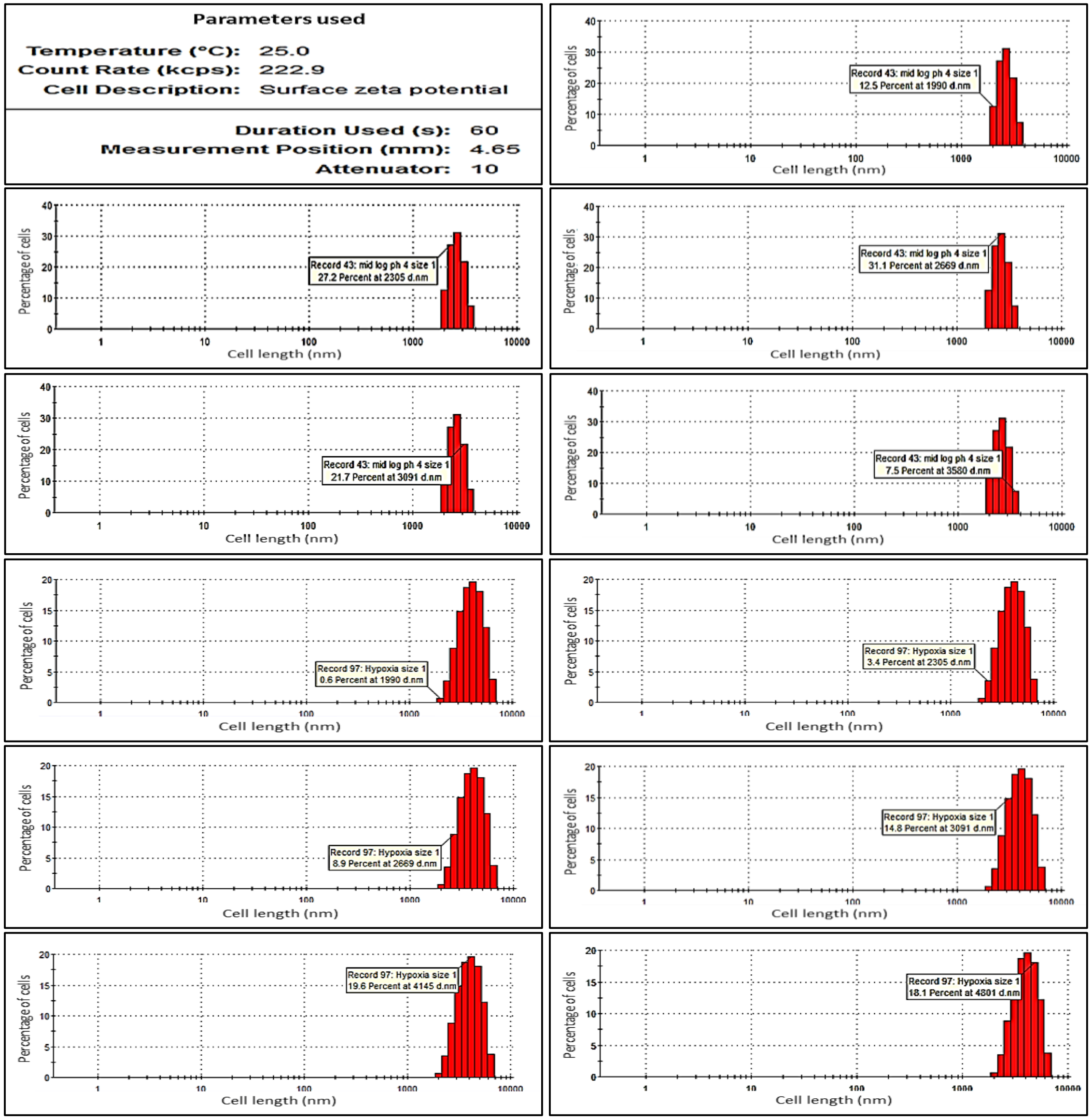

Figure S1. Size Measurement using Dynamic Light Scattering (DLS) using the Instrument, Malvern Zetasizer nano ZS (Sensitivity - 0.3 nm - 10 µm). **(A)**. Average lengths of NRP stage 2 cells and the proportion of cells of such lengths. **(B)**. Average lengths of MLP cells and the proportion of cells of such lengths. **(C & D)**. Cell length distribution of MLP and NRP stage 2 cells determined using Zetasizer. Each peak represents cell size (nm) and the percentage of cells at that particular cell size range.

A

MLP		
**Expected region (cm^{-1})**	**Observed values (cm^{-1})**	**IR Stretching frequency (cm^{-1})**
3650-3200	3229	OH, Broad peak Hydrogen-bond
3400-2400	2357	OH (intermolecular) stretching frequency
1350-1000	1065	C-N frequency
970-700	976	Trans disubstituted alkenes

NRP-II		
**Expected region (cm^{-1})**	**Observed values (cm^{-1})**	**IR Stretching frequency (cm^{-1})**
3650-3200	3276	Broad peak Hydrogen-bonded
2926	2935	CH_2 of methine frequency
1680-1630	1649	C=O of amide frequency
1640-1550	1545	N-H of amide bend frequency
1350-1000	1033	C-N frequency
970-700	976	Trans disubstituted alkenes

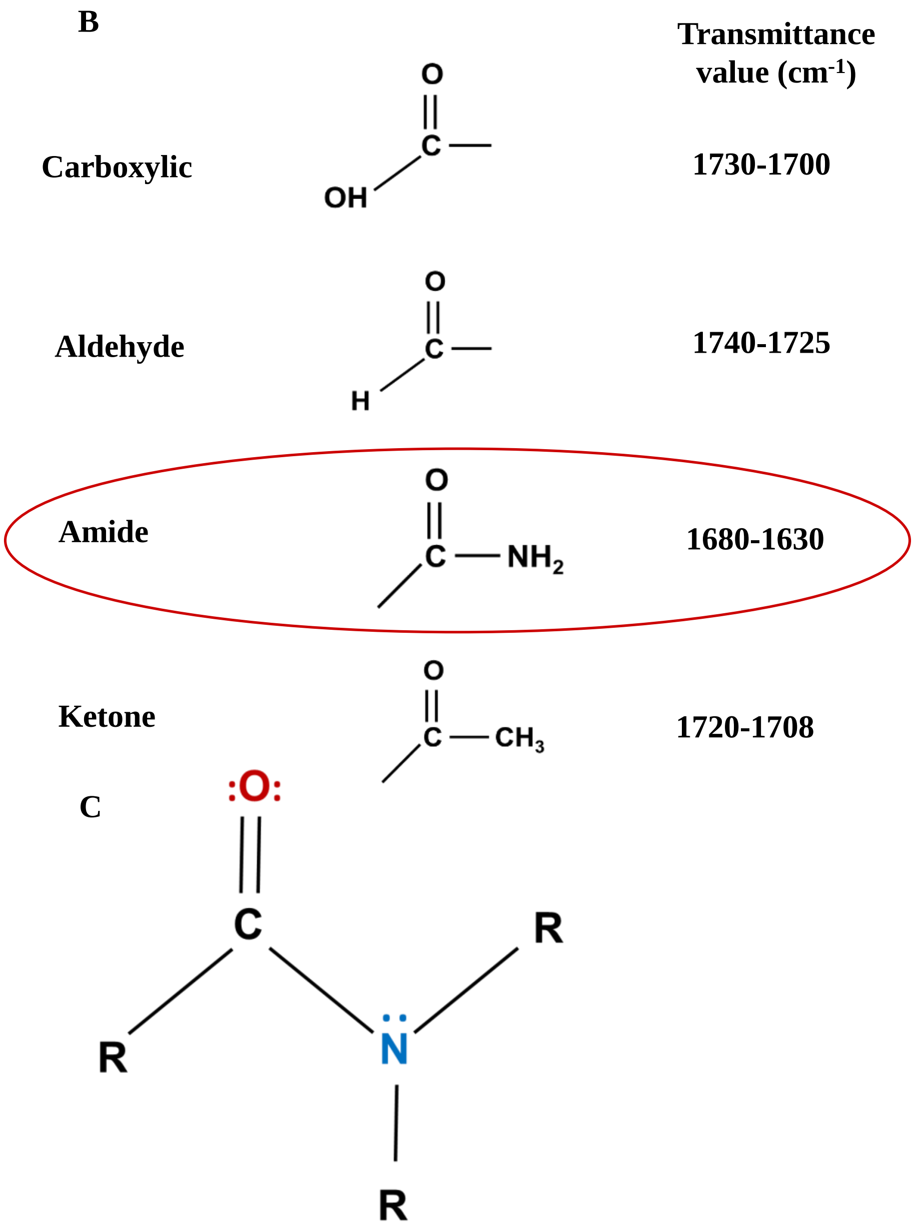

Figure S2. (A) Major functional group differences between *Mtb* MLP and NRP stage 2 cells from FTIR analysis. (**B**) Most probable pairing of carbonyl group with other functional groups. **(C)** Diagrammatic representation of carbonyl group with amide bend frequency [Pavia et al., 2001].

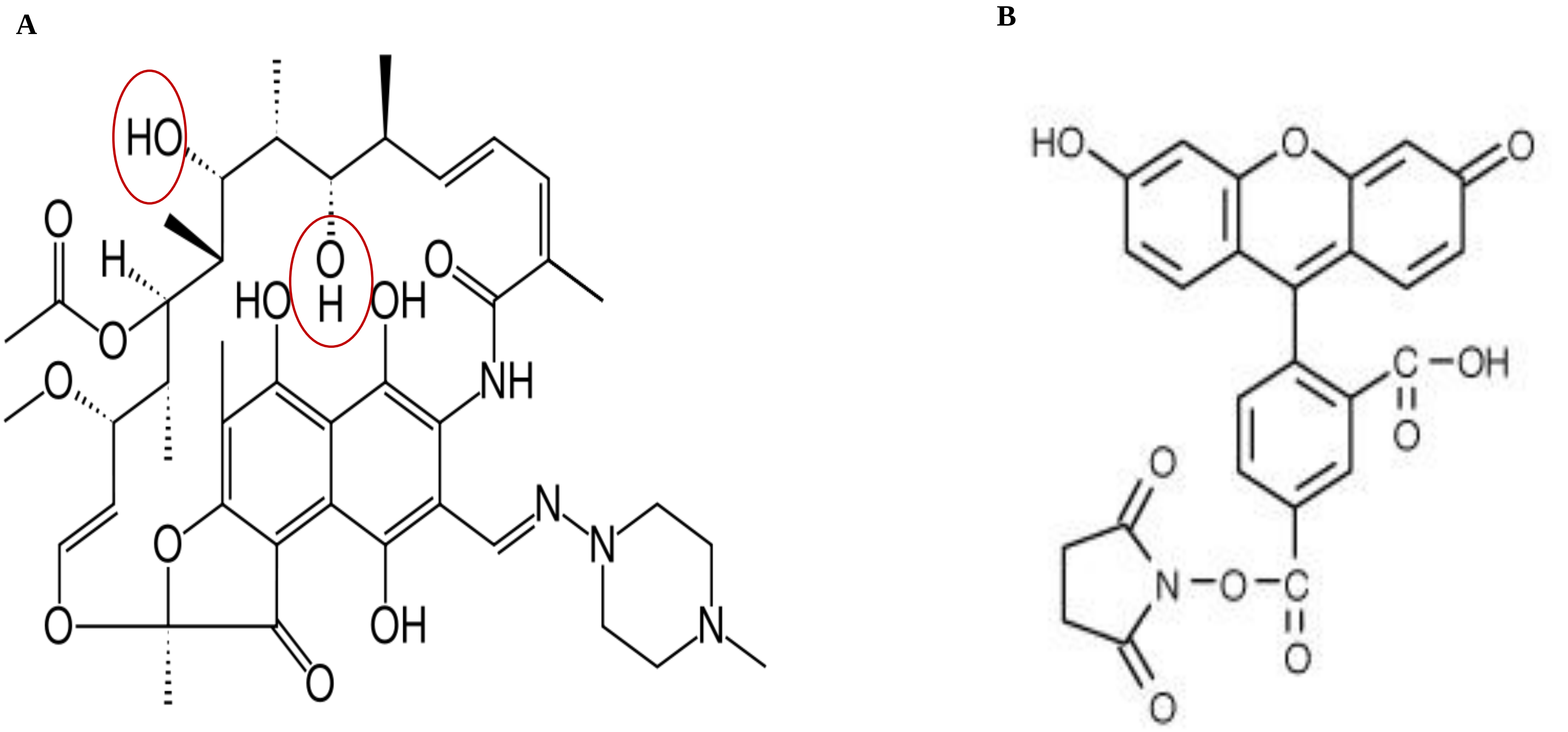

Figure S3. Chemical structures of the antibiotic (Rifampicin) and the fluorophore 5- carboxyfluorescein (5-FAM). **(A)**. Chemical structure of the antibiotic Rifampicin. **(B)**. The conjugate 5-corboxy fluorescein (5-FAM). Conjugation site for 5-FAM on rifampicin is encircled in red **(A)**.

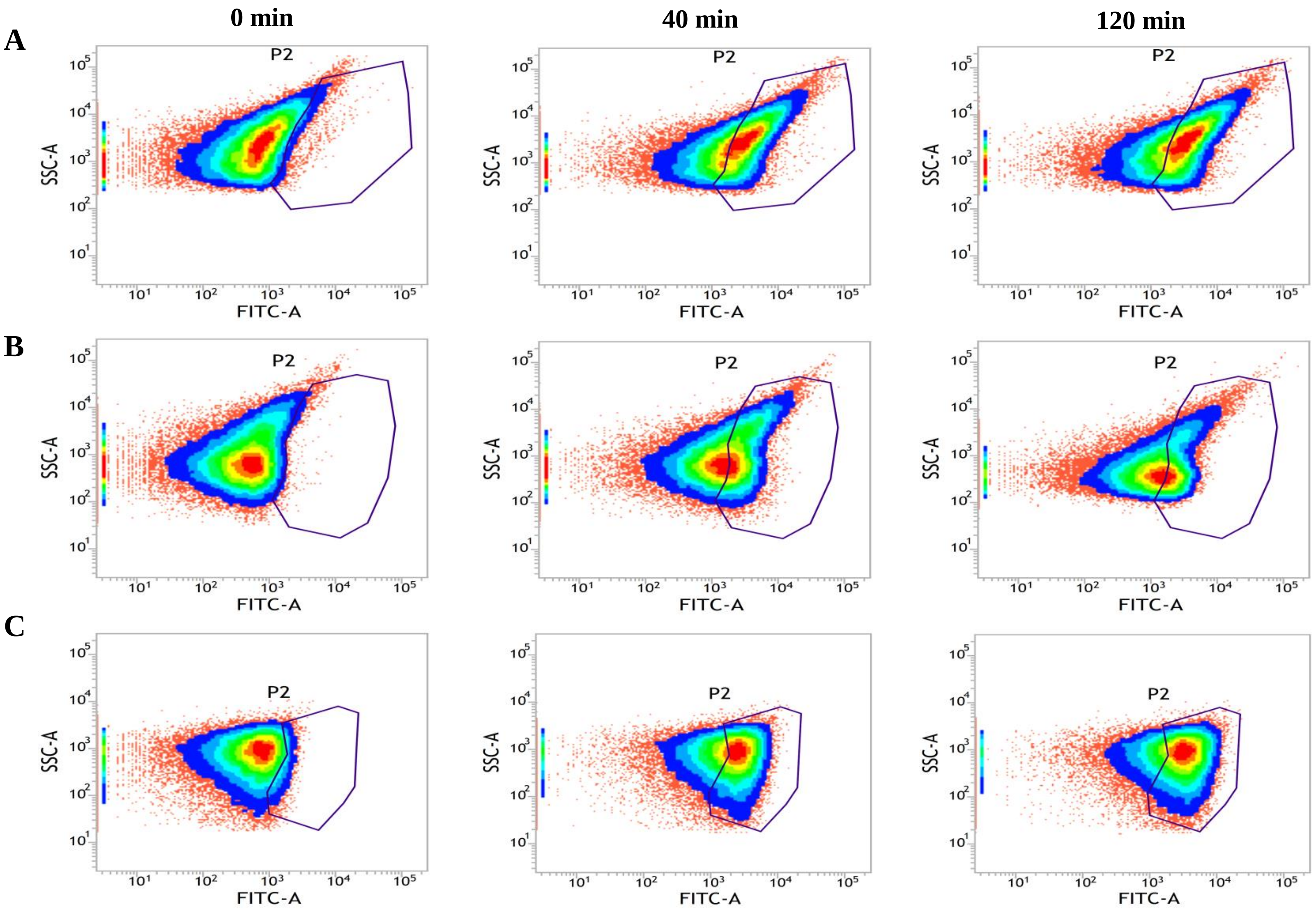

Figure S4. Flow cytometry profile of the 5-FAM fluorescence as a measure of the permeability of 5-FAM-RIF into *Mtb* MLP and NRP stage 2 cells over a period of 120 min. **(A)** MLP cells; **(B)** NRP stage 2 cells; **(C)** NRP stage 2 bead-beaten cells.

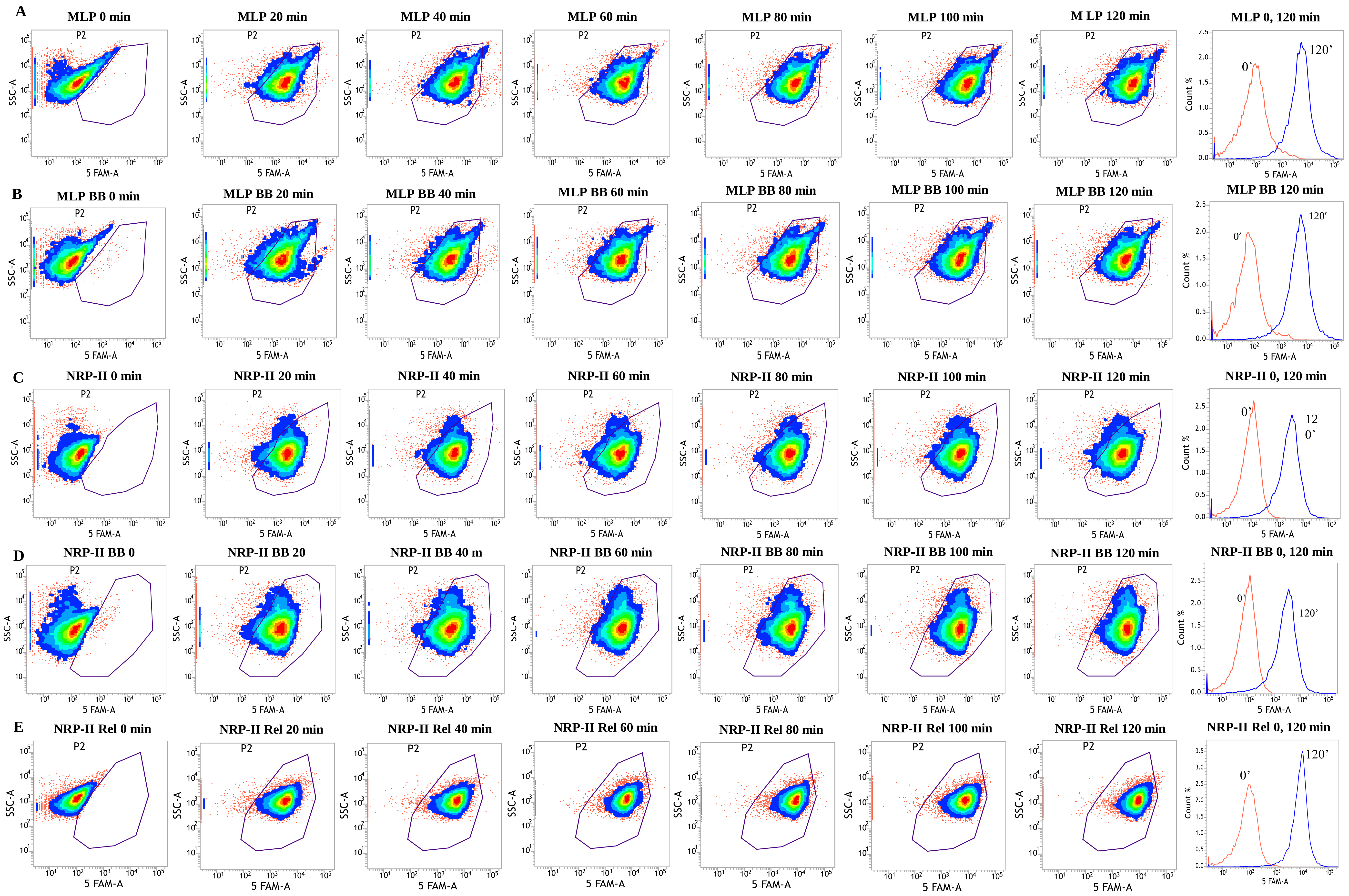

Figure S5. Flow cytometry profile of the 5-FAM fluorescence as a measure of the permeability of 5-FAM-RIF into *Mtb* MLP and NRP stage 2 cells over a period of 120 min. **(A)** MLP cells; **(B)** MLP bead-beaten cells; **(C)** NRP stage 2 cells; **(D)** NRP stage 2 bead-beaten cells; **(E)** NRP stage 2 cells post-release from hypoxia into normoxia.

Table S1. Oligonucleotides used for qRT-PCR in the study

Name	Oligonucleotide sequence	Purpose
Mtb-otsB1-RT-f	5’ – attggtcgggcacagttgat – 3’	qRT-PCR
Mtb-otsB1-RT-r	5’ – gacttctatctcggcggtgg – 3’	qRT-PCR
Mtb-galE2-RT-f	5’ – gatgttcaccgaggacagca – 3’	qRT-PCR
Mtb- galE2-RT-r	5’ – agtcactgcaatatcggggc – 3’	qRT-PCR
Mtb-pimB -RT-f	5’ – gcggcttcgttggaatacct – 3’	qRT-PCR
Mtb-pimB -RT-r	5’ – caaccgcgacagacacacta – 3’	qRT-PCR
Mtb-LdtA-RT-f	5’ – agtgggtcgctagcaatgtc – 3’	qRT-PCR
Mtb-LdtA-RT-r	5’ – aggtatgtgccgagatgctg – 3’	qRT-PCR
Mtb-glgB-RT-f	5’ – caacgactccgccaacaatg – 3’	qRT-PCR
Mtb-glgB-RT-r	5’ – cagcccgagtcgatagtcac – 3’	qRT-PCR
Mtb-malQ-RT-f	5’ – gttgtcgtcggtgaggatct – 3’	qRT-PCR
Mtb-malQ -RT-r	5’ – cgcaatctcgatcctgctca – 3’	qRT-PCR
Mtb-udgA-RT-f	5’ – accgtatcgtccttggggta – 3’	qRT-PCR
Mtb-udgA-RT-r	5’ – tggcggataccttgaccaac – 3’	qRT-PCR
Mtb-LdtB-RT-f	5’ – gatgtggcggtcaacaccta – 3’	qRT-PCR
Mtb-LdtB-RT-r	5’ – cgcacggtcagtatcttggt – 3’	qRT-PCR
Mtb-rpiB-RT-f	5’ – ccaattgatcggcatcggc – 3’	qRT-PCR
Mtb-rpiB-RT-r	5’ – gtgggtccgttcgtactcg – 3’	qRT-PCR
Mtb-1635-RT-f	5’ – ggagtctgctgggccatatc – 3’	qRT-PCR
Mtb-1635-RT-r	5’ – gaagccgcgaccataattcg – 3’	qRT-PCR
Mtb-ponA2-RT-f	5’ – ggatctagaagccggcgaaa – 3’	qRT-PCR
Mtb-ponA2-RT-r	5’ – acgtcgagttgggcgttaat – 3’	qRT-PCR
Mtb-0648-RT-f	5’ – cggcctgttgacccattaca – 3’	qRT-PCR
Mtb-0648-RT-r	5’ – gtcagcgcgacctttttgag – 3’	qRT-PCR
Mtb-ald-RT-f	5’ – cggatccacactcgctactc – 3’	qRT-PCR
Mtb-ald-RT-r	5’ – acctggtttcatatgcgcga – 3’	qRT-PCR
